# Predicting protein–carbohydrate binding sites: a deep learning approach integrating protein language model embeddings and structural features

**DOI:** 10.1093/bib/bbag008

**Published:** 2026-01-29

**Authors:** Md Muhaiminul Islam Nafi, M Saifur Rahman

**Affiliations:** Department of CSE, BUET, Palashi Road, Dhaka 1000, Dhaka District, Bangladesh; Department of CSE, United International University (UIU), United City, Madani Avenue, Badda, Dhaka 1212, Dhaka District, Bangladesh; Department of CSE, BUET, Palashi Road, Dhaka 1000, Dhaka District, Bangladesh

**Keywords:** computational biology, machine learning, prediction, proteins, structural bioinformatics

## Abstract

Protein–carbohydrate interactions play an important role in many biological processes and functions, like inflammation, signal transduction, and cell adhesion. In our work, we will study non-covalent carbohydrate binding sites. In this paper, we aim to build a deep-learning model to predict non-covalent protein–carbohydrate binding sites. We were motivated by the fact that experimental approaches for predicting these sites are expensive. So, computational tools are necessary for identifying these interactions. We explored several sequence-based features as well as structural features. We also leveraged protein language model embeddings. We analyzed different architectures and selected the most suitable deep learning architecture for our finalized prediction model, DeepCPBSite. DeepCPBSite is an ensemble model that combines three separate models with three approaches (random undersampling, weighted oversampling, and class-weighted loss) built on the ResNet+FNN architecture. We made separate datasets from three sources: RCSB, UniProt, and CASP. We also compared the structural features extracted from the structures predicted by AlphaFold and ESMFold in the context of our prediction tasks. We employed three different feature selection techniques and finally did a SHAP (SHapley Additive exPlanations) analysis on the structural features after categorizing the proteins based on their organism information. DeepCPBSite achieved 78.7% balanced accuracy and 59.6% sensitivity on the TS53 set, outperforming the second-best competitor, DeepGlycanSite, by 1.16% and 2.94%, respectively. Additionally, its F1, MCC, and AUPR scores outperformed other state-of-the-art methods, with improvements ranging from 3.77%–47.6%, 3.84%–32.7%, and 8.18%–60.21%, respectively.

## Introduction

Proteins are essential macromolecules that play a vital role in all biological processes happening across various species. Proteins serve several important factors like enzymatic functions, transport and storage, hormonal effects, immune system, cell-to-cell communications, regulation of gene expressions, structural functions, etc. Protein–carbohydrate interaction is also a crucial branch of study that has an influence on several biological functions, like inflammation, signal transduction, cell adhesion, host–pathogen recognition, stabilization of protein structure, etc. Protein–carbohydrate interactions are of two kinds. One is covalent binding, which is a posttranslational modification named glycosylation, and the other is non-covalent binding. In this study, we focus on non-covalent carbohydrate binding sites. If any amino acid in a protein is located within a 3.5 Å distance from any carbohydrate in a protein–carbohydrate complex, the protein is said to be carbohydrate bound, and that interaction between protein and carbohydrate is called protein–carbohydrate binding site [1]. Experimental methods for identifying carbohydrate–protein binding sites are expensive. So, computational prediction methods are required for these tasks. We propose a deep learning (DL) approach for this prediction task in our study.

Taroni *et al*. [2] pioneered a method that predicted protein–carbohydrate binding sites using known protein structures. Six attributes were examined, and the three best ones were chosen. Sujatha and Balaji [3] proposed a tool named COTRAN that was able to detect galactose-binding sites with a good specificity and sensitivity. By assuming common principles for substrate recognition, they tried to characterize a unique galactose-binding signature by analyzing the 3D structures of 18 galactose–protein complexes. Kulharia *et al*. [4] introduced a method named InCa-SiteFinder for predicting non-covalent inositol and carbohydrate binding sites on protein surfaces. Considering the sugar-binding pocket as a spherical spatio-chemical environment, Nassif *et al*. [5], made a vector of geometric and chemical features like charges, hydrophobicity, and hydrogen bonding. Tsai *et al*. [6] built a prediction model by an encoding scheme based on 3D probability density maps. Shanmugam *et al*. [7] identified and analyzed the residues involved in both the folding and binding of the protein–carbohydrate complexes. Malik *et al*. [1] designed a neural network-based prediction model. The features used were binding propensities of each of the 20 residue types, structural features like solvent accessibility, packing density, and secondary structure (SS), and evolutionary features like position-specific scoring matrix (PSSM). Pai *et al*. [8] proposed MOWGLI, an ensemble of base classifiers, for predicting Mannose-interacting residues. The base classifiers were trained with the Random Forest algorithm. The features were evolutionary-based features like PSSM. Agarwal *et al*. [9] created a support vector machine (SVM) based model to predict Mannose interacting residues. They used binary profile of patterns and PSSM. Taherzadeh *et al*. [10] designed SPRINT-CBH, an SVM model to predict carbohydrate binding sites. They used sequence-based features, evolutionary features (PSSM), sequence-derived structural information, physicochemical properties (PHYSICO), protein disorder region, and protein length. Gattani *et al*. [11] proposed StackCBPred, a predictor trained by stacking-based machine learning (ML) methods. SVM, Logistic Regression (LR), K-Nearest Neighbors (KNN), and Extra Tree Classifier were the base classifiers, and SVM was the meta-classifier. The features were evolutionary features (PSSM) and several predicted structural features. Canner *et al*. [12] built two models, CAPSIF:V and CAPSIF:G, that used protein structures for making predictions. CAPSIF:V was a 3D-UNet voxel-based neural network model, and CAPSIF:G was an equivariant graph neural network model. They used structural features generated from protein structures for protein–carbohydrate binding site prediction. Bibekar *et al*. [13] made PeSTo-Carbs, a geometric transformer model, that predicts protein–carbohydrate binding sites using protein structures. They had proposed two models. One is PS-G, a generalized predictor for a broad spectrum of protein–carbohydrate interactions, and another one is PS-S, which was trained on nonhomologous protein structures associated with carbohydrate monomers. He *et al*. [14] made DeepGlycanSite, a deep equivariant graph neural network model with the transformer architecture, that uses geometric and evolutionary features from structures of proteins to predict protein–carbohydrate binding sites.

While the upstream feature generation, such as PLM embeddings, is computationally expensive for both the proposed model and some state-of-the-art (SOTA) methods like DeepGlycanSite, our approach employs a simplified downstream prediction model. Such simplification is crucial for efficient training and fast inference, making our model more practical despite the costly feature extraction step. A training set was generated from one source, while multiple test sets were derived from three separate sources. The ground-truth annotations for all sets have been carefully curated and made publicly available. These annotated datasets will support future comparisons of models and contribute to ongoing research in this field. Recent studies have often neglected the importance of proper feature selection by analyzing different feature groups and exploring appropriate imbalance handling techniques. In contrast, our study systematically handles these aspects by thoroughly analyzing feature groups and employing useful imbalance-handling methods.

In this study, we have introduced a novel model, DeepCPBSite, to predict carbohydrate binding sites using a ResNet+FNN architecture, where one branch incorporates ResNet-based convolutional layers and the other branch consists of a feedforward neural network (FNN). We curated datasets from three sources: RCSB [15], UniProt [16], and CASP [17]. We have used protein language model (PLM) embeddings (ProtT5-XL-U50 [18], ESM-2 [19]) and structural features. We employed three separate feature selection techniques: Incremental Feature Selection (IFS), Recursive Feature Elimination (RFE), and Elastic Net [20]. We analyzed five different dataset imbalance handling methods: random undersampling, weighted oversampling, class-weighted loss, SMOTE [21], and ADASYN [22]. We also generated results using structural features extracted from both experimental structures (retrieved from RCSB) and predicted structures (generated by AlphaFold [23] and ESMFold [19]) at inference time. The models were trained solely on features derived from experimental structures, but during evaluation, we tested their performance using structural features from predicted structures. Notably, when comparing the predictive performance on AlphaFold- and ESMFold-predicted structures, the ESMFold-based inputs yielded slightly better results. We conducted a SHAP (SHapley Additive exPlanations) [24] analysis on the structural features to evaluate the importance of these features within each kingdom/category for both RCSB and UniProt datasets, generating dedicated plots for each kingdom/category. We further used SHAP on the ResNet branch to investigate the contribution of local residue context to the model predictions. We added a case study that demonstrates DeepCPBSite’s performance in diverse structural environments.

The key contributions of this work can be summarized as follows.

- We have outperformed all SOTA methods for protein–carbohydrate binding site prediction. Our proposed model is architecturally simpler and more efficient.
- We have constructed datasets from three sources: RCSB, UniProt, and CASP.
- We employed three different feature selection techniques—IFS, RFE, and Elastic Net—to select the optimal feature set for our prediction task. All three methods yielded the same feature set, indicating a strong consensus and highlighting the robustness of the selected features.
- We have explored different traditional ML and DL architectures and found ResNet+FNN deep learning architecture to be the most suitable candidate.
- Five different imbalance handling approaches were analyzed and compared in our study. Among these, three techniques—random undersampling, weighted Oversampling, and class-weighted loss—were selected for the final ensemble model.
- We have compared AlphaFold- and ESMFold-predicted structures for the prediction of non-covalent carbohydrate binding sites and concluded that ESMFold-predicted structures perform slightly better than AlphaFold-predicted ones.
- We have made a SHAP analysis on the structural features for different kingdoms/categories belonging to the two types of datasets (RCSB and UniProt). To find out how local residue context affects model predictions, we also performed SHAP analysis on the ResNet branch.

The sections can be summarized as follows: Section materials and  methods describes the datasets, feature extraction processes, feature selection methods, performance metrics, the design of the model architecture, and model configurations. Section Results presents the performance evaluation, results of different experiments, and comparative investigation of our approach, and outlines the SHAP analysis on the structural features and ResNet branch. Section discussion and conclusion explores the essence of our discoveries in the context of non-covalent carbohydrate–protein binding site prediction and contains future directions.

## Materials and methods

### Dataset

In this section, datasets extracted from RCSB, UniProt, and CASP databases are described. We built one training set and two test sets from RCSB. Additionally, we constructed one test set from UniProt and one test set from CASP. Each dataset was carefully annotated, and the ground truths have been made available publicly.

#### Datasets retrieved from RCSB

We used RCSB [15] for gathering proteins that have oligosaccharides as the branch identity type and have no glycosylations in it. We did an advanced search in RCSB with search keywords as follows:

QUERY: ( Branched Entity Type = "oligosaccharide"

AND Glycosylation Site NOT = "C-Mannosylation"

AND Glycosylation Site NOT = "N-Glycosylation"

AND Glycosylation Site NOT = "O-Glycosylation"

AND Glycosylation Site NOT = "S-Glycosylation" )

AND Polymer Entity Type = "Protein"

This yielded 4029 proteins. The proteins that did not have structures available were discarded. We produced protein chain-wise target values. For each protein chain, there was a separate FASTA. Only proteins possessing more than five carbohydrate-binding residues (residues with one or more heavy atoms within 3.5 Å of any carbohydrate atom) were included in our selection. We also ignored any chain that had nonstandard amino acids, as we cannot get the PHYSICO of the nonstandard amino acids. We considered 101 identified HET (heterogeneous) molecules as carbohydrates that were given in Procarb [25]. A total of 5213 protein--carbohydrate chains were thus obtained. With a 30% pairwise sequence similarity limit, we used BLAST-CLUST [26] to further eliminate redundant proteins. A total of 774 protein--carbohydrate complex chains were thus obtained. Some of the residues from the FASTA sequences were unmodeled or partially modified in some proteins. We took the FASTAs of each protein chain from RCSB and made them aligned with residues obtained from protein structure files using the Needleman--Wunsch algorithm [27]. The gap (‘-’) in the alignment, as annotated in our dataset file, represents the residues from the original FASTA that were unmodeled. The target values of the unmodeled residues were also represented as gaps (‘-’). There were 771 proteins with 774 protein chains. There were 3323 41 residues in total, and among them, 3140 17 were modeled from the original FASTA. Among them, 8498 residues were carbohydrate--protein binding (positive) sites, and 3055 19 residues were nonbinding (negative) sites. We call this the *RCSB dataset*.

We randomly selected about 80% of the proteins as the *training dataset* and about 20% of the proteins as the *independent test set*. We overall conserved the positive and negative ratios in both training and independent datasets. The training set had 622 protein chains, and the independent set had 152 protein chains. The 152 protein chains of the independent set belonged to 152 distinct proteins. To make comparative performance analysis among our method and DeepGlycanSite [14] and CAPSIF [12], we further excluded from this independent set any protein that had >30% pairwise sequence similarity with the training sets of those methods, using BLAST-CLUST [26]. We did not consider PeSTo-Carbs’s training set for this filtering due to dataset inconsistencies between their paper and GitHub repositories. Furthermore, to ensure structural-level dissimilarity, we also removed proteins that exhibited fold-level homology to the training set. After these filters, a set of 53 nonredundant proteins remained, which we refer to as the *TS53 set*. Thus, one training and two test sets were generated from RCSB. We did an 80-20 split on the training set, with the former (referred to as the training-80 set) being used for training, and the latter (referred to as the validation set) being used for model validation. In the experiments related to model selection, we trained the models on the training-80 set and tested them on the validation set.

#### Dataset retrieved from UniProt

We only generated a test set from the proteins retrieved from UniProt [16]. The keywords that were used for the advanced search were as follows:

(ft_binding_exp:"CHEBI:16646") AND (reviewed:true)

NOT (ft_carbohyd:s-linked) NOT (ft_carbohyd:c-linked)

NOT (ft_carbohyd:o-linked) NOT (ft_carbohyd:n-linked)

We extracted 125 proteins from the advanced search. We then used BLAST-CLUST [26] with a 30% sequence identity limit. After excluding proteins with $\leq 5$ carbohydrate-binding residues, 53 proteins remained. We again ran BLAST-CLUST [26] to reduce the sequence identity with the training set to <30%. Finally, we got 37 proteins containing 409 positive sites and 15458 negative sites. In total, there were 15867 sites. We named this dataset *TS37 set*.

#### Dataset retrieved from CASP

For broader applicability, we collected a dataset from CASP16 [17]. We took the protein complexes that had experimental structures available and used the same filters that we used for the RCSB dataset. We have ensured 30% pairwise sequence similarity limit from the training set. Also, we removed proteins that exhibited fold-level homology to the training set. After the filtering, we got 11 protein complexes that contained 14 protein chains. We refer to this new dataset as the *TS14 set*. The TS14 set was constructed entirely from CASP16 targets to ensure independence from RCSB and Uniprot databases. Therefore, these data provide an ideal benchmark for the evaluation of our model’s generalization capability beyond familiar data distributions. We use the TS14 set to further validate our proposed model under this domain-shifted setting, thereby providing a more vigorous test of its robustness and wider applicability. The datasets constructed from RCSB, UniProt, and CASP are summarized in Table 1.

**Table 1:** Summary of the datasets prepared from RCSB, UniProt, and CASP

Source	Dataset	Positive sites	Negative sites	Total sites	Protein chains	Total protein chains	Total proteins
RCSB	Training	6704	241 805	248 509	622	774	771
	Independent	1794	63 714	65 508	152		
	TS53	649	24 276	24 925	53	53	53
UniProt	TS37	409	15 458	15 867	37	37	37
CASP	TS14	74	5349	5423	14	14	11

### Feature extraction

The feature groups used in our study are described in this subsection.

#### Position-Specific Scoring Matrix

PSSM is a matrix that shows the conservation of residues at each place in a sequence alignment. It is used for adding evolutionary information to sequence alignments. Evolutionary information has been proven effective in many biological analysis and prediction tasks [28--33]. We used PSI-BLAST [34] for extracting PSSM features from protein sequences. We set the parameters ‘‘inclusion_ethresh’’ to 0.001 and ‘‘num_iterations’’ to 3 during the extraction. The feature group size of PSSM is 20 for each residue.


(1) \begin{align*}& PSSM = \begin{bmatrix} P_{1,1} & P_{1,2} & \dots & P_{1,20} \\ \vdots & \vdots & \vdots & \vdots \\ P_{L,1} & P_{L,2} & \dots & P_{L,20} \end{bmatrix}\end{align*}

In equation 1, L is the sequence length of a protein, and 20 is the column size representing 20 amino acids (AA).

#### Dipeptide composition

Dipeptide composition (DPC) [35] captures the interactions between amino acid residues across different positions. At first, the $20\times 20$ matrix was calculated for each residue in the protein sequence and then flattened to get a feature group size of 400.


(2) \begin{align*}& DPC(k,i,j)= \begin{cases} P_{k,i} P_{k+1,j} & \textrm{if}\ k=1 \\ P_{k,i} P_{k-1,j} + P_{k,i} P_{k+1,j} & \textrm{if}\ 1<k<L \\ P_{k,i} P_{k-1,j} & \textrm{if}\ k=L \end{cases}\end{align*}

In equation 2, k represents the residue position within a protein sequence, $P_{k,i}$ represents the ith column cell of the PSSM matrix of the kth residue, $P_{k+1,j}$ represents the jth column cell of the PSSM matrix of the (k+1)th residue and $P_{k-1,j}$ represents the jth column cell of the PSSM matrix of the (k-1)th residue. DPC produced a matrix of (L, 20, 20), and after flattening, it became (L, 400).

#### Monogram

Monogram (MG) [36, 37] feature was calculated from the PSSM matrix using equation 3. The feature group size was 1.


(3) \begin{align*}& MG(k) = \sum_{i=1}^{20} P_{k,i}\end{align*}

#### Physicochemical properties

We used seven different physicochemical features (polarizability, steric parameter, isoelectric point, hydrophobicity, helix probability, normalized Van der Waals volume, and sheet probability) for each of the 20 standard amino acids. The values used are identical to the values used in [38]. The feature group size was 7.

#### ProTrans models

The authors of ProtTrans [18] investigated four auto-encoder models (BERT, Albert, Electra, T5) and two auto-regressive models (Transformer-XL, XLNet) by using 393 billion amino acids of UniRef and BFD data. The available 10 models are ProtT5-XL-BFD, ProtT5-XL-UniRef50 (or ProtT5-XL-U50), ProtT5-XXL-BFD, ProtT5-XXL-UniRef50, ProtBert, ProtBert-BFD, ProtXLNet, ProtAlbert, ProtElectra-Discriminator-BFD, and ProtElectra-Generator-BFD. Among them, ProtT5-XL-U50 performed the best in TS115, CASP12, DeepLoc, and CB513. ProtAlbert has an embedding size of 4096, while all others have an embedding size of 1024. We produced per-residue embedding for seven models, excluding ProtT5-XL-BFD, ProtT5-XXL-BFD, and ProtT5-XXL-UniRef50 due to our resource constraints.

#### ESM-2

Through the use of masked language modeling, ESM2 [19], which is a transformer-based PLM, was trained on millions of different natural proteins during evolution. For unsupervised self-attention contact map predictions, it gives per-residue embeddings, and it was trained to identify the structural implications of interconnected sequence patterns. We used the esm2_t33_650M_UR50D model. From it, we extracted per-residue embeddings of size 1280.

#### ProteinBERT

ProteinBERT [39], a deep language model, was created to naturally capture both local and global representations of proteins. This model was trained on about 106 million proteins obtained from UniProtKB/UniRef90. We extracted a per-residue embedding of size 128 for each residue.

#### TAPE

TAPE [40] provided some pre-trained models for generating residue-wise embeddings of size 768. We generated per-residue embeddings from one of its models, named ‘‘bert-base,’’ for our study.

#### Structural features (Structural)

Motivated by [41], we incorporated a number of structural features in our study. The size of this feature group was 36. We used one-hot encoding for SS, which were Alpha helix, Isolated beta-bridge residue, Strand, 3-10 helix, Pi helix, Turn, Bend, None, and Irregular SS. We divided relative solvent accessibility (RSA) values into 10 bins: $ \textrm{RSA} \leq 0.1 $, $ 0.1 < \textrm{RSA} \leq 0.2 $, $ 0.2 < \textrm{RSA} \leq 0.3 $ and so on, up to $ 0.9 < \textrm{RSA} \leq 1 $. We also used Phi and Psi angles. We used DSSP [42] to extract these features from the protein structure files.

The other features were neighbor counts around 12 Å, virtual surface area, relative sequential and spatial positioning, three vectors, bond angle between C==O, and bond angle between CA-CA. Virtual surface area is the area of the convex hull created by the atoms of the residue. The sequential position feature was represented by the inverse of the index of the residue in the protein sequence. The spatial position feature was represented by the inverse of the spatial distance between the residue and the centroid of the protein. When calculating the centroid coordinates, we calculated the mean of the positional vectors of the center atoms across all residues. Among the three vectors, one was a vector from the center atom of the ith residue toward that of the (i-1)th residue. The second one was a vector from the center atom of the ith residue toward that of the (i+1)th residue. And the last one was a vector from the alpha carbon toward the beta carbon. Lastly, the angle between C==O of the ith residue and C==O of the (i-1)th residue, and the angle between CA-CA of the (i-1)th and ith residue, and CA-CA of the ith and (i+1)th residue were used. The summary of structural features is given in Table 2.

**Table 2:** Description of structural features

L, Size	Description
L, 9	One hot encoding for SS
L, 10	RSA bins
L, 1	Phi angle
L, 1	Psi angle
L, 1	Neighbor counts around 12 Å
L, 1	Virtual surface area
L, 2	Relative positioning
L, 9	Three vectors
L, 1	Bond angle between C==O
L, 1	Bond angle between CA-CA

#### Word embedding

We took our inspiration to use word embedding in this work from [43]. Firstly, We numbered the 20 amino acids and gap, ‘‘ARNDCQEGHILKMFPSTWYV-,’’ from 0 to 20. For each target residue, we considered a window of 15 downstream and 15 upstream residues. This 31-sized sequence was passed through PyTorch’s [44] embedding layer to generate the word embedding. This was used as input for the ResNet branch to utilize local patterns among neighboring residues.

### Feature selection

The Yeo--Johnson transformation [45] was performed on the feature groups to make data more Gaussian-like. For RFE and Elastic Net [20], we used functions from the python package *scikit-learn*. The 10 ML models used for the 10fold CV performance analysis were: XGBoost (XGB), Extra Trees (ET), Random Forest (RF), Partial Least Squares (PLS), KNN, Decision Tree (DT), SVM, Multi-Layer Perceptron (MLP), LR, and Naive Bayes (NB). Three feature selection techniques were employed to enhance the robustness of the feature selection process.

#### Incremental feature selection

Out of the feature groups we examined, we chose the five top groups (based on their 10-Fold CV F1-score) and ran incremental feature selection (IFS) on those as follows. First the feature group with the highest F1-score is taken. Then we try to add one other feature group to it and measure the prediction performance. Of all the feature group pairs thus created, we keep the pair that has the best 10-fold CV F1-score. Now we try to add another feature group to this pair in the same way. In this way, instead of exploring all possible feature group combinations exhaustively, we explored a subset of them in a greedy manner to obtain the optimal feature group combination. The optimal feature group combination turned out to be ‘‘ProtT5-XL-U50, Structural, ESM-2’’ (see Results). To conduct IFS, SVM was used as the learning algorithm because of its superior 10-fold CV performance compared with other learners.

#### Recursive feature elimination

RFE is a popular method for selecting features in prediction tasks. In RFE, a wrapper model is fitted recursively, features are ranked according to relevance, and less significant features are discarded one at a time. The process is repeated until the specified number of features is obtained. We chose XGB as the learning algorithm for running RFE on the top five feature groups. We set the number of features to be selected to 100. It produced a rank for each feature. After that, we calculated the feature count from each group within the top 100 features. The score for each feature group was calculated using equation 4.


(4) \begin{align*}& RFE{\_}Score = \frac{\text{Number of features of the group in top 100}}{\sqrt{\textrm{Total number of features in the group}}}\end{align*}

The score is normalized by the square root of feature group size (instead of the size directly) so that large feature groups are not overly penalized. Based on these scores, the combination of ‘‘ProtT5-XL-U50, Structural, ESM-2’’ was selected (see Results).

#### Elastic Net

L1 (Lasso) and L2 (Ridge) penalties are combined in Elastic Net [20]. The combination of L1 and L2 regularization helps it identify the best features. In our study, Elastic Net gave 660 features from the top five feature groups. We gave a score to each feature group using equation 5.


(5) \begin{align*}& \textrm{Elastic}{\_}\textrm{Net}{\_}\textrm{Score} = \frac{N_{g}}{N_{s} \sqrt{F_{g}}} ,\end{align*}

where $N_{g}$ is the number of selected features of the group, $N_{s}$ is the total number of selected features, and $F_{g}$ is the total number of features in the group.

As in the case of $RFE{\_}Score$, the square root was used in the normalizing divisor, not to over-penalize larger feature groups. In this approach too, the combination of ‘‘ProtT5-XL-U50, Structural, ESM-2’’ was selected (see Results).

### Performance metrics

We used several reputed metrics [46--49] for the evaluation of different models and features during our study. We utilized Area Under the Precision-Recall Curve (AUPR), Precision (PREC) (also known as Positive Predictive Value), F1 Score (F1) (the harmonic mean of Precision and Recall), Specificity (SP) (also known as True Negative Rate), Sensitivity (SN) (also known as Recall or True Positive Rate), Balanced Accuracy (BACC) (the average of Sensitivity and Specificity), Matthews Correlation Coefficient (MCC), Accuracy (ACC), and Area Under the ROC (Receiver Operating Characteristic) Curve (AUC).


(6) \begin{align*} &\ \ \ \ \ \ \ \ PREC = \frac{TP}{FP+TP}\qquad\qquad\qquad\qquad\qquad\qquad\quad\qquad\qquad\qquad\ \ \end{align*}

(7) \begin{align*} & F1 = \frac{2TP}{2TP+FP+FN}\qquad\qquad\qquad\qquad\qquad\qquad\quad\ \qquad\quad\ \ \ \end{align*}

(8) \begin{align*} & SP = \frac{TN}{TN+FP}\ \qquad\qquad\qquad\qquad\qquad\qquad\quad\ \qquad\qquad\qquad\ \ \end{align*}

(9) \begin{align*} & SN = \frac{TP}{TP+FN}\qquad\qquad\qquad\qquad\qquad\qquad\qquad\qquad\qquad\qquad\, \end{align*}

(10) \begin{align*}\ \ \ \ \ \ \ & BACC = \frac{SN+SP}{2}\qquad\qquad\qquad\qquad\qquad\qquad\qquad\qquad \qquad \ \ \ \ \ \end{align*}

(11) \begin{align*}\ \ & MCC = \frac{(TP \times TN) - (FP \times FN)}{ \sqrt{(TP + FN) \times (TP + FP) \times (TN + FP) \times (TN + FN)}} \end{align*}

(12) \begin{align*} & ACC = \frac{TP+TN}{FP+TP+TN+FN}\qquad\qquad\qquad\qquad\qquad\qquad\quad\ \ \end{align*}

In equations 6, 7, 8, 9, 10, 11, and 12, TP, FP, TN, and FN represent True Positives, False Positives, True Negatives, and False Negatives, respectively.

### Model selection

We executed a 10-fold CV on the whole training set, balanced using random undersampling. We saw that a simple FNN performed better than the traditional ML models with respect to AUPR and AUC. So, the DL approach was proven to be suitable for our prediction task. We then compared the simple FNN with ResNet+FNN and Transformer+FNN trained on the training-80 set and tested on the validation set. The inputs of the ResNet branch of the ResNet+FNN model and the transformer branch of the Transformer+FNN model were word embeddings. The inputs of the FNN branch were the optimal feature set chosen from feature selection techniques. ResNet+FNN significantly improved the performance compared with the simple FNN by capturing the local patterns among the neighboring residues. We sent our selected feature set from feature selection to the FNN branch and word embedding to the ResNet branch. So, we concluded the ResNet+FNN architecture as our final model.

### Handling imbalanced dataset

The dataset has far more negative samples than positives. To handle this severe imbalance, we utilized five strategies: random undersampling, weighted oversampling, class-weighted loss, SMOTE [21], and ADASYN [22]. In random undersampling, we took all positive samples and randomly chose the same number of negative samples. In the case of weighted oversampling, a datapoint is sampled with probability inversely proportional to the proportion of its class frequency. Class-weighted loss assigns greater weight to the minority class loss than the majority class, in inverse proportion to the class frequency. New synthetic samples are generated by SMOTE (Synthetic Minority Over-sampling Technique). It interpolates between minority samples and their neighbors. ADASYN (Adaptive Synthetic Sampling) is another oversampling technique that gives more priority to the minority class samples that are harder to learn. This strengthens the model’s ability to discern difficult situations. We measured the performance of each approach on the validation set and found models trained using random undersampling, weighted oversampling, and class-weighted loss approaches to be suitable for making an ensemble model (see Results).

### Model architecture

The models trained with random undersampling, weighted oversampling, and class-weighted loss are denoted as $M_{RU}, M_{WO}, \textrm{ and}\ M_{CWL}$, respectively. All three different models ($M_{RU}$, $M_{WO}$, and $M_{CWL}$) had the same architecture.

#### ResNet branch

Here, we gave the word embedding as input. We used one embedding layer and three consecutive ResNet blocks in this branch. Inside the ResNet block, there were two conv1d layers. At first word embeddings had the size of (Batch_size, 31). After going through the embedding layer, it became (Batch_size, 31, 21). The embedding layer internally kept a lookup table where rows were indexed from 0 to 20. The weights inside the table were updated throughout the training process. After that, it was permuted to make the input suitable for conv1d processing. The three ResNet blocks each contained two conv1d layers that were followed by a maxpool1d layer. Each of the conv1d layers had a 1D filter of size 3 (kernel size 3), padding of 1, and a stride value of 1. The number of filters for each layer was 10. Max pooling was done after each ResNet block to only pass the meaningful features and discard the less significant ones. We employed a slightly different approach compared with the original ResNet architecture. We concatenated the input and output tensors rather than performing element-wise addition in each ResNet block. The first ResNet block produced an output tensor of size (Batch_size, 31, 31) from the input tensor of size (Batch_size, 21, 31), and after max-pooling, it became a tensor of the shape (Batch_size, 31, 15). Similarly, the second ResNet block produced a (Batch_size, 41, 15) shaped output tensor that was reduced to (Batch_size, 41, 7) after maxpooling. Finally, after going through the third ResNet block and then max-pooling, the size of the output tensor became (Batch_size, 51, 3). After the ResNet processing, the size became (Batch_size, 51, 3). This tensor was then flattened and fed to a linear layer. The linear layer outputs a tensor with the shape (Batch_size, 32). This branch recognized the local patterns between neighboring residues that significantly contributed to the last prediction.

#### Feedforward neural network branch

Here, the inputs were ProtT5-XL-U50 embedding, structural features, and ESM-2 embedding. The architecture had two linear layers. The first linear dense layer received the input of size 2340 and output a tensor of shape (Batch_size, 512). Lastly, the second linear layer output a tensor of size (Batch_size, 256).

**Figure 1.**
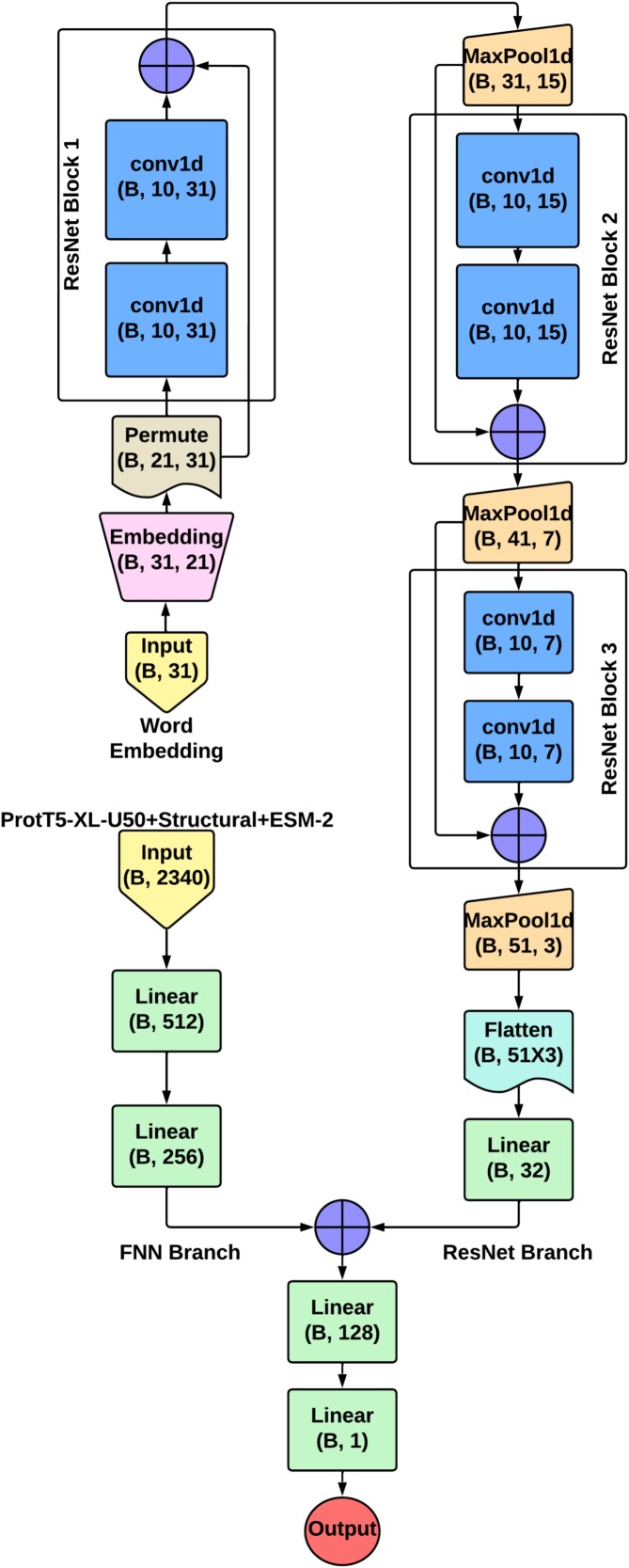
Architecture of the ResNet+FNN model consisting of a ResNet branch with three residual blocks processing word embeddings and a fully connected neural network branch processing the optimal feature set, where “B” denotes Batch_size and output tensor dimensions are shown within each block.

**Figure 2.**
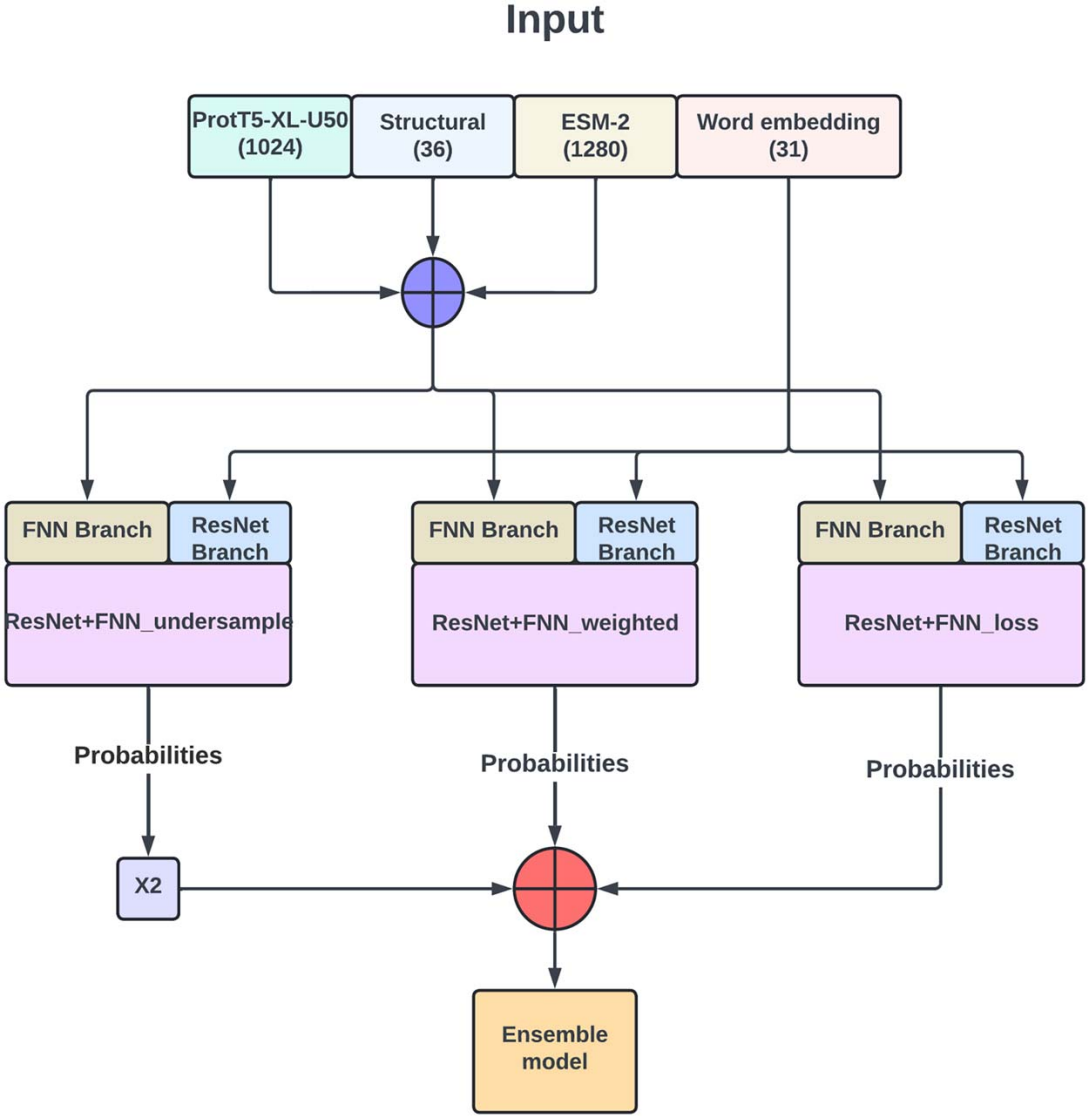
The architecture for the final ensemble model (DeepCPBSite). Here, three separate models ($\textit{M}_{\textit{RU}}$, $\textit{M}_{\textit{WO}}$, and $\textit{M}_{\textit{CWL}}$), which are defined as ‘’ResNet+FNN_undersample,” “ResNet+FNN_weighted,” and “ResNet+FNN_loss,” respectively, are combined. The probabilities for each model are generated, with ResNet+FNN_undersample’s probabilities assigned double the weight of the others. The weighted average of these probabilities is the final probability of the ensemble model.

The outputs of these two branches were concatenated and then fed to the final two linear layers. We used this architecture to build three separate models based one the aforementioned three data imbalance handling strategies. The three models were then combined into an ensemble. The ResNet+FNN architecture is shown in Fig. 1, and the ensemble architecture in Fig. 2. The class discriminating threshold used throughout the experiments was set to 0.5 for all 10 ML models and other DL models. After each linear dense layer, we applied batch normalization, dropout, and ReLU activation. ReLU was used to bring nonlinearity to the model. Batch normalization and dropout were employed to improve convergence and generalization. The hyperparameter configuration of the final model is given in Table 3.

**Table 3:** Hyperparameter configuration of the final model

Parameters	Values
Input feature size (FNN branch)	2340
Input feature size (ResNet branch)	31
Activation function (linear layers and conv1ds)	ReLU
Activation function (output layer)	Sigmoid
Optimizer	Adam
Loss function	Binary cross-entropy loss
Initial learning rate	0.00001
Dropout	0.5
Batch size	2048
Batch normalization	1D
Kernel size (conv1d)	3
Kernel size (maxpool1d)	2
Padding (conv1d)	1
Stride (conv1d)	1
Dilation (conv1d)	1
Output channels (conv1d)	10
Epoch (random undersampling)	180
Epoch (weighted oversampling)	100
Epoch (class-weighted loss)	325

## Results

### ProtT5-XL-U50 provides the best embedding for the non-covalent carbohydrate--protein binding site prediction

We trained the 10 ML classifiers on the training set represented by each PLM embedding. Random undersampling was used to balance the training set. For each PLM embedding, 10-fold CV performance, averaged over all the classification algorithms, is reported in Table 4. ProtT5-XL-U50 embeddings performed the best among all ProTrans model embeddings, and ESM-2 embeddings performed best among the other ones across almost all metrics. So both these embeddings were used as features.

**Table 4:** 10-fold CV performance of all PLM embeddings, averaged over all 10 ML classifiers trained on the randomly undersampled training dataset

Feature	SN	SP	BACC	ACC	PREC	F1	MCC	AUC	AUPR
ProtAlbert	0.728	0.729	0.732	**0.794**	0.720	0.729	0.460	0.775	0.738
ProtBert	0.726	0.739	0.732	0.732	0.739	0.730	0.467	0.802	0.787
ProtBert-BFD	0.729	0.766	0.747	0.747	0.759	0.742	0.497	0.818	0.805
ProtElectra-Discriminator-BFD	0.707	0.756	0.731	0.731	0.742	0.722	0.464	0.794	0.780
ProtElectra-Generator-BFD	0.707	0.756	0.731	0.731	0.742	0.722	0.464	0.794	0.780
ProtT5-XL-U50	**0.774**	**0.804**	**0.789**	0.789	**0.800**	**0.785**	**0.579**	**0.859**	**0.849**
ProtXLNet	0.690	0.702	0.696	0.696	0.697	0.691	0.393	0.760	0.738
ESM-2	0.748	0.790	0.769	0.769	0.782	0.763	0.540	0.835	0.823
ProteinBERT	0.703	0.685	0.694	0.694	0.691	0.697	0.388	0.756	0.736
TAPE	0.717	0.705	0.711	0.711	0.709	0.713	0.423	0.776	0.757
ProtT5-XL-U50-Window	0.786	0.711	0.748	0.748	0.732	0.757	0.499	0.810	0.775
ESM-2-Window	0.742	0.704	0.723	0.723	0.714	0.727	0.447	0.784	0.748

We also considered a window with 15 residues on either side of the target site. For each of these residues, we extracted the per-residue embedding. For residues near the N-terminus or C-terminus, embeddings were extracted only for the residues present within the windows, ignoring any missing positions. We then averaged these vectors to generate the window embeddings. The rows in Table 4 with ‘‘ProtT5-XL-U50-Window" and ‘‘ESM-2-Window" in the Feature column represent the performance of this approach with the PLM being ProtT5-XL-U50 and ESM-2, respectively. As can be observed, the windowing approach degraded the performance for both language models. We hypothesize that the per-residue embeddings in both models already capture rich contextual information about each residue, and averaging over a window potentially dilutes important local distinctions, reducing model performance.

#### Structural, PSSM, and DPC are the best feature groups apart from PLM embeddings

For the rest of the feature groups, the 10-Fold CV and the results are shown in Table 5. PSSM, DPC, and structural features performed better than the others based on AUPR, F1, and MCC scores. Therefore, we got our top five feature groups: ProtT5-XL-U50, ESM-2, structural, PSSM, and DPC.

**Table 5:** 10-Fold CV performance of feature groups other than PLM embeddings, averaged over all 10 ML classifiers trained on the randomly undersampled training dataset

Feature	SN	SP	BACC	ACC	PREC	F1	MCC	AUC	AUPR
PSSM	0.629	0.705	0.667	0.667	0.682	0.654	0.336	0.725	0.715
Monogram	0.408	**0.751**	0.579	0.579	0.640	0.473	0.174	0.605	0.617
DPC	0.616	0.724	0.670	0.670	**0.691**	0.649	0.343	0.726	0.719
PHYSICO	0.619	0.663	0.641	0.641	0.584	0.600	0.283	0.670	0.641
Structural	**0.696**	0.681	**0.689**	**0.689**	0.688	**0.691**	**0.378**	**0.747**	**0.732**

#### SVM was selected to be used with IFS, and XGB with RFE

After determining the top five feature groups, we trained 10 ML classifiers with the training set featurized using these feature groups. Each classifier was trained with each feature group separately. Then we averaged the performance of each classifier across the feature groups. The results are reported in Table 6. SVM performed the best, and XGB came second based on almost all metrics. Therefore, we used SVM with the IFS feature selection technique. However, SVM with ‘‘rbf’’ kernel does not work with RFE in the python scikit-learn library. Hence, we went with XGB for running RFE.

**Table 6:** 10-fold CV performance of all 10 ML models averaged over the top five feature groups. Training was conducted on the randomly undersampled training dataset. The highest metrics are boldfaced, and the second-highest metrics are underlined

Classifier	SN	SP	BACC	ACC	PREC	F1	MCC	AUC	AUPR
XGB	**0.733**	0.758	0.745	0.745	0.752	0.742	0.491	0.821	**0.821**
ET	0.690	0.790	0.740	0.740	0.768	0.726	0.483	0.813	0.816
RF	0.703	0.782	0.742	0.742	0.764	0.732	0.487	0.817	0.819
PLS	0.689	0.751	0.720	0.720	0.733	0.709	0.441	0.791	0.789
KNN	0.699	0.693	0.696	0.696	0.693	0.695	0.394	0.757	0.725
DT	0.640	0.638	0.639	0.639	0.639	0.640	0.279	0.640	0.590
SVM	0.725	**0.792**	**0.759**	**0.759**	**0.775**	**0.749**	**0.519**	**0.829**	**0.821**
MLP	0.731	0.732	0.732	0.732	0.731	0.731	0.463	0.801	0.796
LR	0.720	0.745	0.732	0.732	0.737	0.728	0.465	0.804	0.799
NB	0.596	0.729	0.662	0.662	0.692	0.634	0.333	0.711	0.703

#### Feature selection results

**Figure 3.**
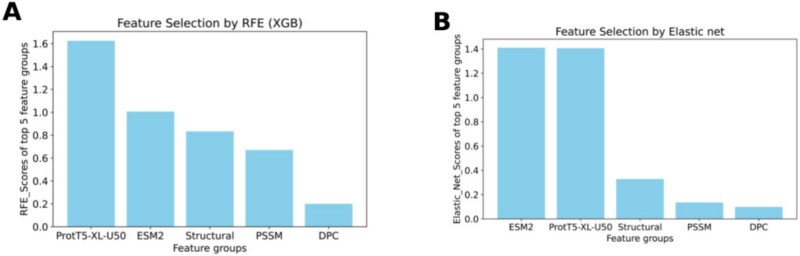
Bar plots of feature selections using RFE with XGB and Elastic Net generated from the scores of each top five feature groups. (A) Bar plot of feature selection using RFE with XGB. (B) Bar plot of feature selection using Elastic Net.

We ran IFS, RFE, and Elastic Net on the top five feature groups. IFS results are shown in Table 7. We can see that SVM models trained using the feature sets ‘‘ProtT5-XL-U50, Structural, ESM-2" and ‘‘ProtT5-XL-U50, Structural, ESM-2, PSSM" have the same F1-score. Considering Occam’s razor principle, we chose the feature set ‘‘ProtT5-XL-U50, Structural, ESM-2" from IFS. The feature selection results from the RFE and Elastic Net approaches are captured in Fig. 3. Here we have generated plots using the scores (mentioned in section feature selection) of feature groups for both RFE and Elastic Net. In both cases, ProtT5-XL-U50, ESM-2 embeddings, and structural features were the top three feature groups. Thus, from all three feature selection techniques, we concluded that ‘‘ProtT5-XL-U50, Structural, ESM-2’’ was the best feature set.

**Table 7:** 10-Fold CV performance of the feature sets using IFS with SVM classifier trained on the randomly undersampled training set. The row of the feature set with the highest F1-score is boldfaced and underlined. The best F1-scores in each phase are boldfaced

Feature set	SN	SP	BACC	ACC	PREC	F1	MCC	AUC	AUPR
ProtT5-XL-U50	0.828	0.843	0.836	0.836	0.841	**0.834**	0.672	0.910	0.903
ESM-2	0.828	0.843	0.836	0.836	0.841	0.834	0.671	0.907	0.903
PSSM	0.637	0.759	0.698	0.698	0.726	0.678	0.399	0.765	0.752
DPC	0.631	0.789	0.710	0.710	0.750	0.685	0.425	0.777	0.769
Structural	0.703	0.724	0.713	0.713	0.718	0.711	0.427	0.784	0.776
ProtT5-XL-U50, ESM-2	0.836	0.850	0.843	0.843	0.848	0.842	0.686	0.917	0.911
ProtT5-XL-U50, PSSM	0.828	0.845	0.836	0.836	0.842	0.835	0.673	0.910	0.904
ProtT5-XL-U50, DPC	0.822	0.839	0.830	0.830	0.836	0.829	0.661	0.904	0.898
ProtT5-XL-U50, Structural	0.837	0.856	0.847	0.847	0.854	**0.845**	0.694	0.917	0.912
**ProtT5-XL-U50, Structural, ESM-2**	**0.844**	**0.857**	**0.850**	**0.850**	**0.855**	**0.849**	**0.701**	**0.922**	**0.916**
ProtT5-XL-U50, Structural, PSSM	0.836	0.855	0.846	0.846	0.852	0.844	0.692	0.917	0.912
ProtT5-XL-U50, Structural, DPC	0.832	0.844	0.838	0.838	0.842	0.837	0.676	0.911	0.906
ProtT5-XL-U50, Structural, ESM-2, PSSM	0.843	0.857	0.850	0.850	0.855	**0.849**	0.700	0.922	0.916
ProtT5-XL-U50, Structural, ESM-2, DPC	0.838	0.851	0.844	0.844	0.849	0.843	0.689	0.919	0.913
ProtT5-XL-U50, Structural, ESM-2, PSSM, DPC	0.838	0.851	0.845	0.845	0.849	**0.844**	0.689	0.919	0.913

### Model selection

In this subsection, the FNN model and traditional ML models were compared first. After that, different DL architectures were examined.

#### Comparison between deep learning and traditional machine learning models

We did a 10-fold CV on the randomly undersampled training set for 10 traditional ML models and a simple FNN model in Table 8. Here, we used the optimal feature set as input features in each of the models. FNN outperforms the traditional ML models based on AUPR and AUC. So, for processing the optimal features, the FNN model is finalized. It also proved that DL models hold promise for this prediction task. Therefore, we further pursued different DL architectures.

**Table 8:** 10-fold CV performance of all classifiers trained on the randomly undersampled training dataset. We calculated the means and standard deviations of the scores across 10 folds. The values are presented in “mean $\pm $ standard_deviation” format

Classifier	AUPR	AUC
XGB	0.909 $\pm $ 0.009	0.909 $\pm $ 0.008
ET	0.887 $\pm $ 0.011	0.883 $\pm $ 0.009
RF	0.884 $\pm $ 0.011	0.880 $\pm $ 0.008
PLS	0.892 $\pm $ 0.012	0.891 $\pm $ 0.008
KNN	0.818 $\pm $ 0.017	0.847 $\pm $ 0.014
DT	0.639 $\pm $ 0.008	0.699 $\pm $ 0.009
SVM	0.916 $\pm $ 0.013	0.922 $\pm $ 0.008
MLP	0.911 $\pm $ 0.013	0.917 $\pm $ 0.010
LR	0.870 $\pm $ 0.018	0.882 $\pm $ 0.012
NB	0.749 $\pm $ 0.017	0.776 $\pm $ 0.013
FNN	**0.919** $\pm $ 0.012	**0.924** $\pm $ 0.008

#### Selection of deep learning architecture

We compared simple FNN with ResNet+FNN and Transformer+FNN by training them on the training-80 set and testing on the validation set. The results are shown in Table 9. As described in section model selection, the optimal feature set selected using feature selection techniques served as the input for the FNN branch. Word embeddings served as the input for both the transformer branch of the Transformer+FNN model and the ResNet branch of the ResNet+FNN model. ResNet+FNN performed significantly better than the other two based on almost all metrics. So, ResNet+FNN was chosen as the final DL architecture. ResNet+FNN outperformed Transformer+FNN because ResNet operates on a local neighborhood, effectively capturing spatial dependencies within the word embeddings. In contrast, Transformers focus on global relationships, which may not provide additional benefits in this context, as the Transformer-based protein embeddings used in the FNN branch already incorporate global information. Adding another Transformer in the secondary branch thus does not contribute significantly to the final prediction, whereas ResNet enhances the local feature extraction, leading to better performance. So, we finalized the ResNet branch for processing Word embeddings, and the FNN branch was chosen for handling the optimal feature set.

**Table 9:** Performance of DL models trained on the training-80 set and tested on the validation set

Model	SN	SP	BACC	ACC	PREC	F1	MCC	AUC	AUPR
FNN	**0.869**	0.841	0.855	0.841	0.131	0.228	0.300	0.923	0.334
ResNet+FNN	0.846	**0.866**	**0.856**	**0.866**	**0.149**	**0.254**	**0.321**	**0.924**	**0.337**
Transformer+FNN	0.778	0.835	0.806	0.833	0.116	0.201	0.258	0.890	0.324

#### Ensemble

Five separate models were trained using five different imbalance handling approaches on the training-80 set. They were tested on the validation set. The results are shown in Table 10. We defined the models trained using random undersampling, weighted oversampling, class-weighted loss, SMOTE, and ADASYN as $M_{RU}, M_{WO}, M_{CWL}, M_{S}\text{, and} M_{A}$, respectively. Among these, $M_{RU}$ achieved the highest sensitivity, indicating superior ability to detect positive instances but had lower precision, F1, and MCC scores. $M_{WO}$ and $M_{CWL}$, despite having lower sensitivity than $M_{RU}$, provided better performance across F1-score, MCC, and AUPR. In contrast, $M_{S}$ and $M_{A}$ exhibited comparatively low sensitivity (0.398 and 0.395, respectively) and did not offer meaningful improvements in balanced accuracy or F1-score compared with $M_{WO}$ and $M_{CWL}$ despite their high specificity. This pattern suggests that synthetic sample generation by SMOTE and ADASYN had introduced noisy or redundant minority (positive) class examples, which led to overfitting on the majority (negative) class boundary and reduced generalization performance. Therefore, these two models were excluded from the ensemble. We selected $M_{RU}$, $M_{WO}$, and $M_{CWL}$---which demonstrated complementary strengths---for ensemble construction. We made an ensemble of these models using a weighted averaging technique where $M_{RU}$ was given twice the weight compared with the other two models, as in equation 1.


(1) \begin{align*}& P(M_{ensemble}) = \frac{2 \cdot P(M_{RU})+P(M_{WO})+P(M_{CWL})}{4},\end{align*}

**Table 10:** Performance of five different models trained on the training-80 set and tested on the validation set

Models	SN	SP	BACC	ACC	PREC	F1	MCC	AUC	AUPR
$M_{RU}$	**0.846**	0.866	**0.856**	0.866	0.149	0.254	0.321	0.924	0.337
$M_{WO}$	0.509	0.987	0.748	0.975	0.530	0.520	0.507	0.918	0.503
$M_{CWL}$	0.548	0.985	0.767	0.974	0.510	**0.528**	**0.515**	0.895	**0.509**
$M_{S}$	0.398	**0.994**	0.696	**0.978**	0.643	0.492	0.496	0.920	0.502
$M_{A}$	0.395	**0.994**	0.695	**0.978**	**0.647**	0.491	0.495	0.920	0.495
$M_{ensemble}$	0.628	0.977	0.802	0.967	0.427	0.509	0.502	**0.933**	0.479

where P() denotes the predicted probability.

The predictive performance of the ensemble is also shown in Table 10. The ensemble model increases precision, F1, and MCC scores compared with $M_{RU}$ and offers higher sensitivity than $M_{WO}$ and $M_{CWL}$. This ensemble, which effectively balances precision and sensitivity, was chosen as the final predictive model and is referred to as **‘‘DeepCPBSite.’’**

### Demonstration of class separation using t-SNE

**Figure 4.**
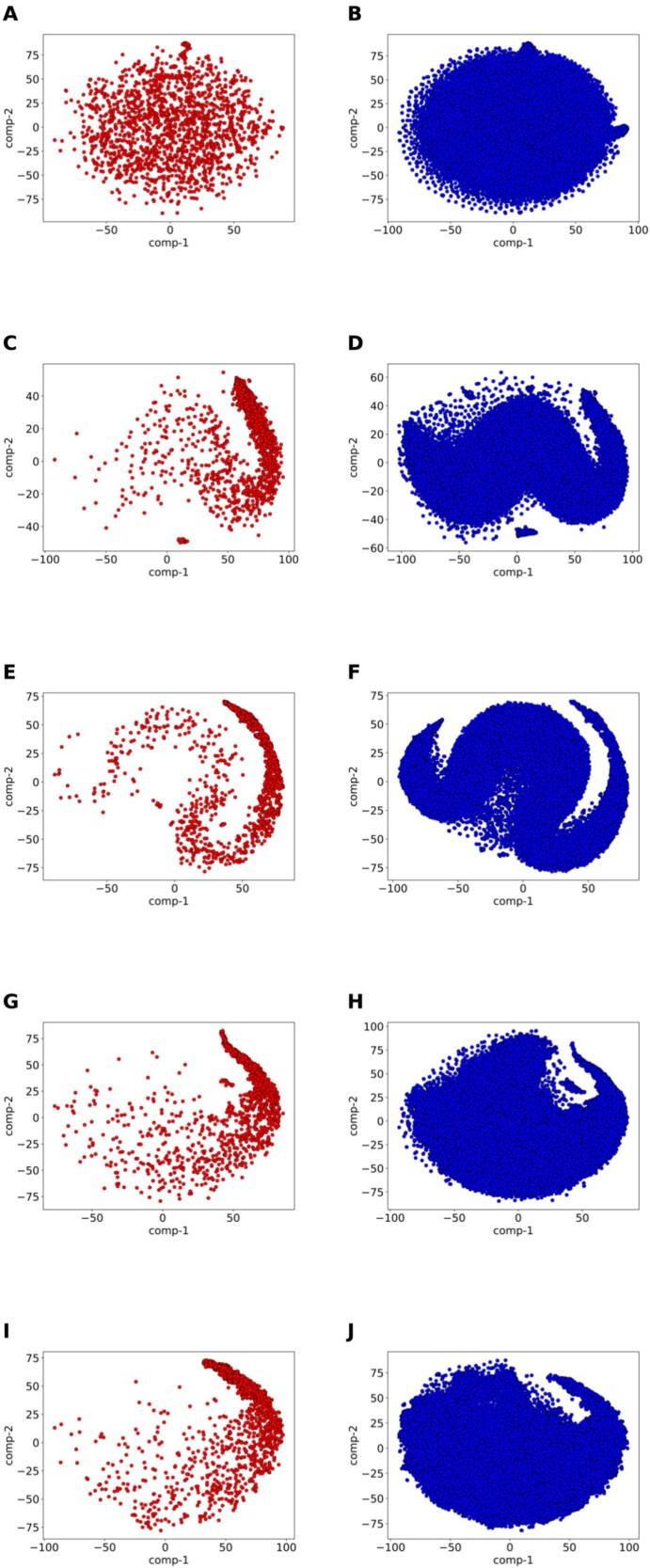
t-SNE visualizations on the independent dataset. (A) t-SNE visualization of initial feature space (positive samples). (B) t-SNE visualization of initial feature space (negative samples). (C) t-SNE visualization of the last hidden layer feature representation of ResNet+FNN random undersampling (positive samples). (D) t-SNE visualization of the last hidden layer feature representation of ResNet+FNN random undersampling (negative samples). (E) t-SNE visualization of the last hidden layer feature representation of ResNet+FNN weighted oversampling (positive samples). (F) t-SNE visualization of the last hidden layer feature representation of ResNet+FNN weighted oversampling (negative samples). (G) t-SNE visualization of last hidden layer feature representation of ResNet+FNN class-weighted loss (positive samples). (H) t-SNE visualization of the last hidden layer feature representation of ResNet+FNN class-weighted loss (negative samples). (I) t-SNE visualization of the last hidden layer feature representation of ResNet+FNN_ensemble (positive samples). (J) t-SNE visualization of the last hidden layer feature representation of ResNet+FNN_ensemble (negative samples).

We used t-distributed stochastic neighbor embedding (t-SNE) [50] to demonstrate how the models separate two different class samples. We created a t-SNE plot to visualize the initial feature space of the samples. Next, we drew three separate t-SNE plots for each model ($M_{RU}$, $M_{WO}$, and $M_{CWL}$) using the feature representations from their respective last hidden layers. Finally, we combined the last hidden layer features from all three models and created a single t-SNE plot to show the overall class separation achieved by the ensemble model. The plots can be found in Fig. 4.

We used all samples from the independent dataset to generate the t-SNE plots demonstrating the class separation. From the plots, we can see that the models reasonably separate the different class samples. The learned feature representations in Fig. 4I and J are more organized than the original feature space (Fig. 4A and B), with the distributions of positive (4I) and negative (4J) samples being more structured. Nonetheless, there is still overlap, indicating that even though the model has made class separability better, it is still challenging to completely differentiate between the two classes. Such overlaps were expected because the dataset is inherently challenging and the classes are not perfectly linearly separable in the feature space. t-SNE plots show a more organized structure: positive samples tend to cluster together, and negative samples form their respective regions. It means that the model learned meaningful embeddings that improve class separability. However, the complete separation remains difficult, showing the intrinsic complexity of the task. t-SNE plots are meant to provide an intuitive visualization of the learned embedding rather than a definitive class separation.

### Performance metrics of DeepCPBSite on the independent set and TS37 set

We tested DeepCPBSite on the independent set and TS37 set in Tables 11 and 12, respectively. Here, two variants of our final model are compared. One is the vanilla DeepCPBSite, and the other is DeepCPBSite without structural features. DeepCPBSite (without struct) has the same architecture, but it does not have the structural features in its FNN branch. The rows labeled DeepCPBSite (AlphaFold) and DeepCPBSite (ESMFold) correspond to the model’s performance using structural features extracted from AlphaFold-predicted structures and ESMFold-predicted structures, respectively, instead of experimental structures.

**Table 11:** Performance of DeepCPBSite on the independent set

Model	SN	SP	BACC	ACC	PREC	F1	MCC	AUC	AUPR
DeepCPBSite	**0.559**	**0.977**	**0.768**	**0.965**	**0.401**	**0.467**	**0.456**	**0.912**	**0.427**
DeepCPBSite (ESMFold)	0.544	0.976	0.760	0.964	0.391	0.455	0.444	0.907	0.412
DeepCPBSite (without struct)	0.532	**0.977**	0.755	**0.965**	0.397	0.455	0.442	0.904	0.407

**Table 12:** Performance of DeepCPBSite on the TS37 set

Model	SN	SP	BACC	ACC	PREC	F1	MCC	AUC	AUPR
DeepCPBSite (AlphaFold)	0.469	**0.983**	0.726	**0.969**	**0.416**	0.441	0.426	**0.913**	**0.376**
DeepCPBSite (ESMFold)	**0.494**	0.982	**0.738**	**0.969**	**0.416**	**0.452**	**0.438**	0.912	0.374

**Figure 5.**
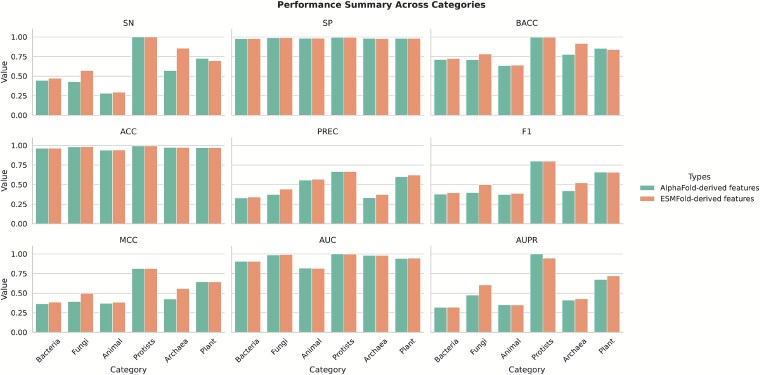
Performance summary across categories for the AlphaFold-derived and ESMFold-derived features. The ESMFold-derived features do better than AlphaFold-derived ones for Fungi and Archaea categories.

For the independent set, we compared DeepCPBSite, DeepCPBSite (ESMFold), and DeepCPBSite (without struct). We did not include DeepCPBSite (AlphaFold) as we could not generate AlphaFold structures for the independent set due to resource constraints. For the TS37 set, we compared DeepCPBSite (AlphaFold) and DeepCPBSite (ESMFold). Performance with ESMFold structure-derived structural features was reasonably better than the performance with AlphaFold structure-derived structural features, in terms of F1, SN, BACC, and MCC scores. One likely explanation is that although ESMFold does not require preexisting databases, AlphaFold substantially relies on them for multiple sequence alignment (MSA) building. ESMFold makes use of language models and DL. To further investigate the matter, we have illustrated a performance summary across categories for the AlphaFold-derived and ESMFold-derived features in Fig. 5. Both features perform almost evenly for all categories except Fungi and Archaea. AlphaFold, which heavily depends on MSA-based evolutionary information and structural templates, performs slightly worse for Fungi and Archaea, where high-quality homologous sequences or structural templates are sparse compared with categories like bacteria and animals. Specifically, since ESMFold is trained directly on protein language representations derived from massive sequence datasets (without relying on explicit MSAs), it generalizes better to underrepresented categories.

We made another analysis with balanced independent and TS37 set in Table 13. We made 20 replicates with all positive samples and randomly chose the same number of negative samples for both datasets. We tested our model, DeepCPBSite, on 20 replicates and measured its performance. The results are shown in ‘‘mean $\pm $ standard_deviation’’ format. Also, we provided the per protein chain (belonging to the independent set and TS37 set) performance results in Supplementary materials.

**Table 13:** Performance of DeepCPBSite on both the independent and the TS37 sets, balanced using random undersampling. Metrics are averaged over 20 replicates

Dataset	SN	SP	BACC	ACC	PREC	F1	MCC	AUC	AUPR
TS37	0.494 $\pm $ 0.000	0.979 $\pm $ 0.008	0.737 $\pm $ 0.004	0.737 $\pm $ 0.004	0.960 $\pm $ 0.015	0.652 $\pm $ 0.004	0.541 $\pm $ 0.012	0.910 $\pm $ 0.006	0.913 $\pm $ 0.009
Independent set	0.559 $\pm $ 0.000	0.978 $\pm $ 0.004	0.768 $\pm $ 0.002	0.768 $\pm $ 0.002	0.962 $\pm $ 0.006	0.707 $\pm $ 0.002	0.591 $\pm $ 0.005	0.913 $\pm $ 0.003	0.918 $\pm $ 0.003

### Comparison with the state-of-the-art methods on the TS53 set

DeepCPBSite outperformed all SOTA methods on the TS53 set in almost all metrics. As mentioned in section datasets retrieved from RCSB, we prepared the TS53 set for comparing our proposed model with DeepGlycanSite and CAPSIF models. We did not consider PeSTo-Carbs in this preparation of the dataset, as their training datasets were not properly given in the provided GitHub repository. Specifically, the number of proteins in the datasets did not match the counts reported by the authors for the training, validation, and test sets of the PS-G model, and the datasets for the PS-S model were not available. We emailed the authors for clarification, but did not get any response from them. We still included PeSTo-Carbs in the comparison using our prepared TS53 set. Despite the possibility of data leakage between PeSTo-Carbs’ actual training set and the TS53 set---potentially inflating its performance---it could not outperform our proposed model in terms of F1 score, MCC, AUC, and AUPR. We ran the provided scripts given by the SOTA methods in their respective GitHub repositories. We generated the probability outputs for all five models, DeepGlycanSite, CAPSIF:V, CAPSIF:G, PS-G, and PS-S. We used experimental structures from RCSB and predicted ESMFold structures during the process.

**Figure 6.**
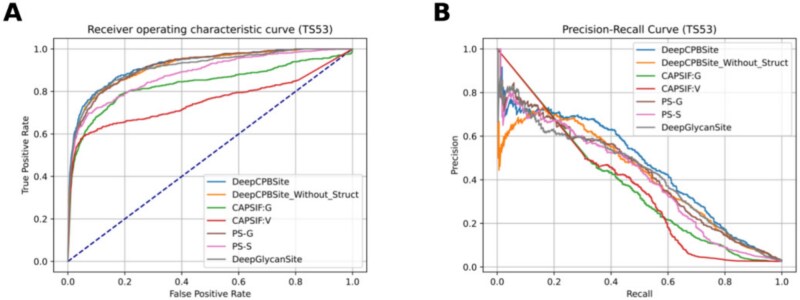
ROC and Precision-recall curves of SOTA methods on the TS53 set using experimental structures. (A) ROC curve. (B) Precision-recall curve.

**Figure 7.**
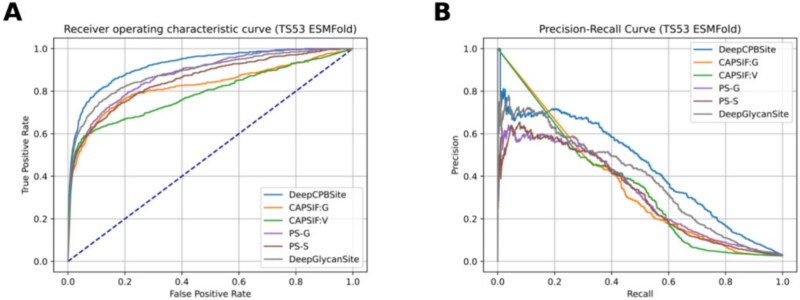
ROC and Precision-recall curves of SOTA methods on the TS53 set using ESMFold structures. (A) ROC curve. (B) Precision-recall curve.

We compared our model with the SOTA models using experimental and ESMFold structures in Tables 14 and 15, respectively. We plotted ROC (Receiver Operating Characteristic) curves and precision-recall curves for each one of them in Figs 6 and 7. In both cases, DeepCPBSite outperformed the existing SOTA methods.

**Table 14:** Comparison of all SOTA methods on the TS53 set using experimental structures

Model	SN	SP	BACC	ACC	PREC	F1	MCC	AUC	AUPR
DeepGlycanSite	0.579	0.977	0.778	0.967	0.406	0.478	0.469	0.915	0.427
CAPSIF:V	0.470	0.981	0.726	0.968	0.402	0.433	0.418	0.754	0.295
CAPSIF:G	0.473	0.977	0.725	0.964	0.358	0.407	0.393	0.835	0.289
PS-G	**0.700**	0.934	**0.817**	0.928	0.221	0.336	0.367	0.919	0.428
PS-S	0.456	**0.988**	0.722	**0.974**	**0.498**	0.476	0.463	0.882	0.405
DeepCPBSite	0.596	0.978	0.787	0.968	0.424	**0.496**	**0.487**	**0.925**	**0.463**
DeepCPBSite (without struct)	0.539	0.980	0.760	0.969	0.420	0.472	0.460	0.918	0.420

**Table 15:** Comparison of all SOTA methods on the TS53 set using ESMFold structures

Model	SN	SP	BACC	ACC	PREC	F1	MCC	AUC	AUPR
DeepGlycanSite	0.545	0.977	0.761	0.966	0.387	0.453	0.442	0.887	0.385
CAPSIF:V	0.468	0.980	0.724	0.966	0.383	0.421	0.406	0.790	0.282
CAPSIF:G	0.435	0.979	0.707	0.965	0.355	0.391	0.375	0.824	0.275
PS-G	**0.584**	0.939	0.762	0.930	0.204	0.303	0.317	0.879	0.323
PS-S	0.376	**0.989**	0.682	**0.973**	**0.468**	0.417	0.406	0.854	0.315
DeepCPBSite	0.572	0.978	**0.775**	0.967	0.405	**0.474**	**0.465**	**0.924**	**0.446**

DeepCPBSite outperformed all other state-of-the-art methods, including DeepGlycanSite, the two CAPSIF variants (CAPSIF:G and CAPSIF:V), and the two PeSTo-Carbs variants (PS-G and PS-S). Although PS-G showed relatively high sensitivity (0.700) and BACC (0.817) when using experimental structures, its precision was significantly low (0.221), resulting in weak F1 (0.336) and MCC (0.367) scores. Similarly, PS-S exhibited inflated specificity (0.988) and precision (0.498) but suffered from low sensitivity (0.456), leading to only moderate F1 (0.476) and MCC (0.463) values. This imbalance suggests the models may be biased toward predicting a particular class. Also, there remains a possibility of data leakage resulting in their metrics being inflated. Despite these inflated metrics in individual categories, neither PS-G nor PS-S surpassed DeepCPBSite in overall performance metrics like F1, MCC, AUC, or AUPR. Notably, DeepGlycanSite emerged as the true second-best method, achieving a better balance across metrics and outperforming the PeSTo-Carbs variants.

DeepCPBSite achieved AUPR scores of 0.463 and 0.446, F1 scores of 0.496 and 0.474, MCC scores of 0.487 and 0.465, and BACC scores of 0.787 and 0.775 on the TS53 set using experimental and ESMFold structures, respectively. It outperformed the second-best model, DeepGlycanSite. DeepCPBSite got 8.43% and 15.84% higher AUPR, 3.77% and 4.64% higher F1, 3.84% and 5.20% higher MCC, and 1.16% and 1.84% higher BACC than DeepGlycanSite when using experimental and ESMFold structures, respectively. Also, we provided the per protein chain (belonging to the TS53 set) performance results in Supplementary materials.

### Comparison with the state-of-the-art methods on the TS14 set

As can be seen from Table 16, DeepCPBSite performs better when compared with all the SOTA methods on the TS14 set. Precisely, it produces the highest precision of 0.233, F1-score of 0.209, MCC of 0.200, and AUPR of 0.176. The relatively high AUC of 0.843 gives further support to its effectiveness in distinguishing binding from nonbinding residues under a domain-shifted evaluation scenario. Nevertheless, the sensitivity of DeepCPBSite is comparatively lower (0.189) than its specificity (0.991). It can be mainly attributed to two factors: (i) substantial class imbalance in the dataset, where the number of positive samples is much fewer than negatives, and (ii) a possible domain shift between the training set derived from RCSB and the TS14 set constructed from CASP16. These findings highlight that domain adaptation remains a challenge in this task, and future studies can explore different techniques to bridge the gap between these two datasets.

**Table 16:** Comparison of all SOTA methods on the TS14 set using experimental structures

Model	SN	SP	BACC	ACC	PREC	F1	MCC	AUC	AUPR
DeepGlycanSite	0.027	0.983	0.505	0.97	0.022	0.024	0.009	0.725	0.026
CAPSIF:G	0	0.99	0.495	0.976	0	0	−0.012	0.52	0.013
CAPSIF:V	0.014	0.99	0.502	0.976	0.018	0.015	0.003	0.587	0.018
PS-G	**0.554**	0.904	**0.729**	0.9	0.075	0.131	0.176	**0.877**	0.07
PS-S	0	**0.994**	0.497	**0.981**	0	0	−0.009	0.627	0.021
DeepCPBSite	0.189	0.991	0.59	0.98	**0.233**	**0.209**	**0.2**	0.843	**0.176**

### Structural feature analysis by SHAP

To categorize the proteins, we followed the three-domain system. The domains are Domain Bacteria, Domain Archaea, and Domain Eukarya. Domain Eukarya is further divided into four kingdoms: Protista, Fungi, Plantae, and Animalia. There are also other categories like viruses, Metagenome, and Synthetic construct (Artificial life entities created by chemical processes). We categorized all proteins using NCBI [51] and GBIF [52] website. The whole categorization is available at Supplementary materials. Some proteins from the RCSB and UniProt datasets were not included as they did not have any organism-specific information. Categorical summaries of RCSB and UniProt datasets are given in Table 17.

**Table 17:** Categorical summary of RCSB and UniProt datasets

Source	Kingdom/Categories	Proteins	Positive sites	Negative sites	Total sites
RCSB	Animal	125	1258	39 558	40 816
	Archaea	11	118	4447	4565
	Bacteria	389	4319	167 666	171 985
	Fungi	54	621	18 155	18 776
	Metagenome	4	54	2456	2510
	Plant	31	327	11 434	11 761
	Protista	9	101	3827	3928
	Synthetic construct	5	46	1612	1658
	Virus	35	379	13 049	13 428
UniProt	Animal	4	85	1280	1365
	Archaea	3	7	460	467
	Bacteria	26	193	7822	8015
	Fungi	3	7	521	528
	Plant	5	33	830	863
	Protista	1	6	498	504

We produced SHAP (SHapley Additive exPlanations) [24] plots for the analysis of structural features of separate kingdoms/categories. We used the function ‘‘GradientExplainer’’ from the python package ‘‘shap’’ to get the SHAP values for all samples. For each category, we took all samples belonging to that category and generated their SHAP values for three different models ($M_{RU}$, $M_{WO}$, and $M_{CWL}$). Then, we concatenated the three arrays containing the SHAP values to generate SHAP bar plots for the ensemble model. The concatenated array was taken as an input to produce the SHAP bar plots.

**Figure 8.**
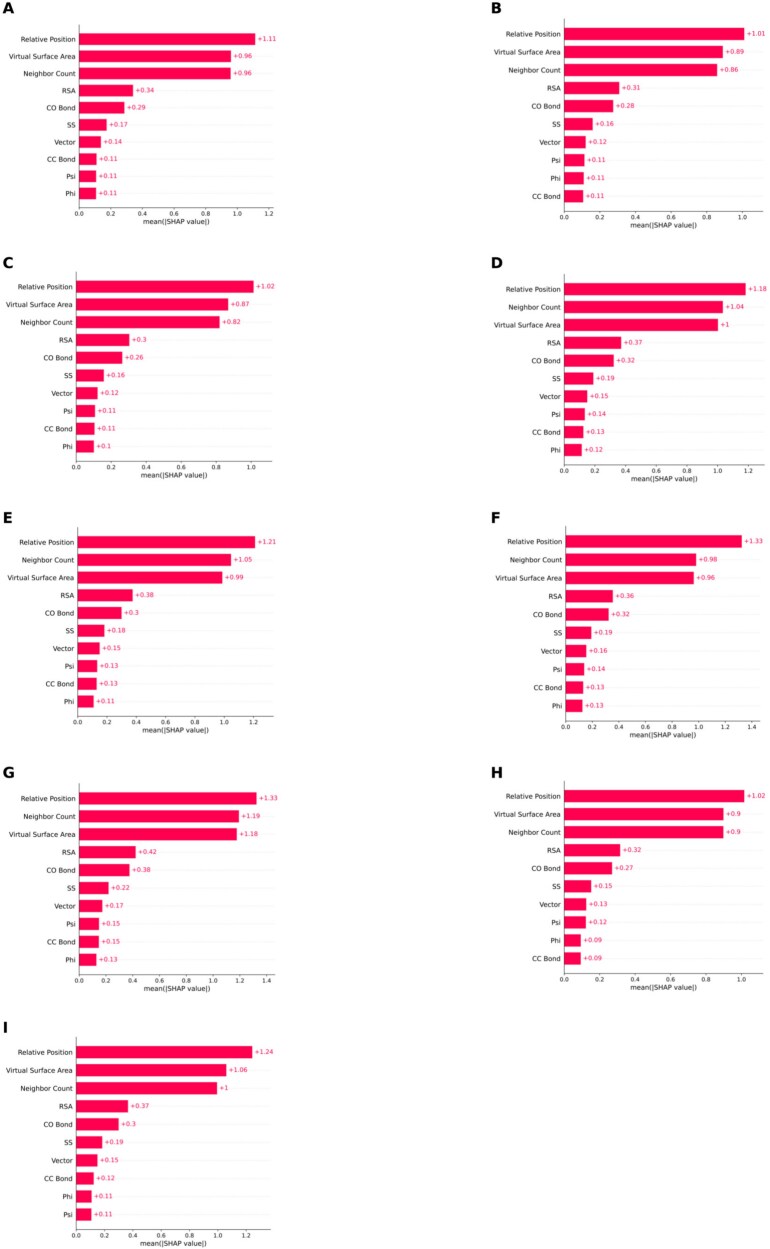
SHAP analysis results for various biological categories in the RCSB dataset. This figure includes the Animal, Archaea, Bacteria, Fungi, Plant, Protista, Virus, Metagenome, and Synthetic construct categories. Each SHAP bar plot was generated from the averaged absolute SHAP values of the structural features. (A) Animal. (B) Archaea. (C) Bacteria. (D) Fungi. (E) Plant. (F) Protista. (G) Virus, (H) Metagenome, (I) Synthetic construct.

**Figure 9.**
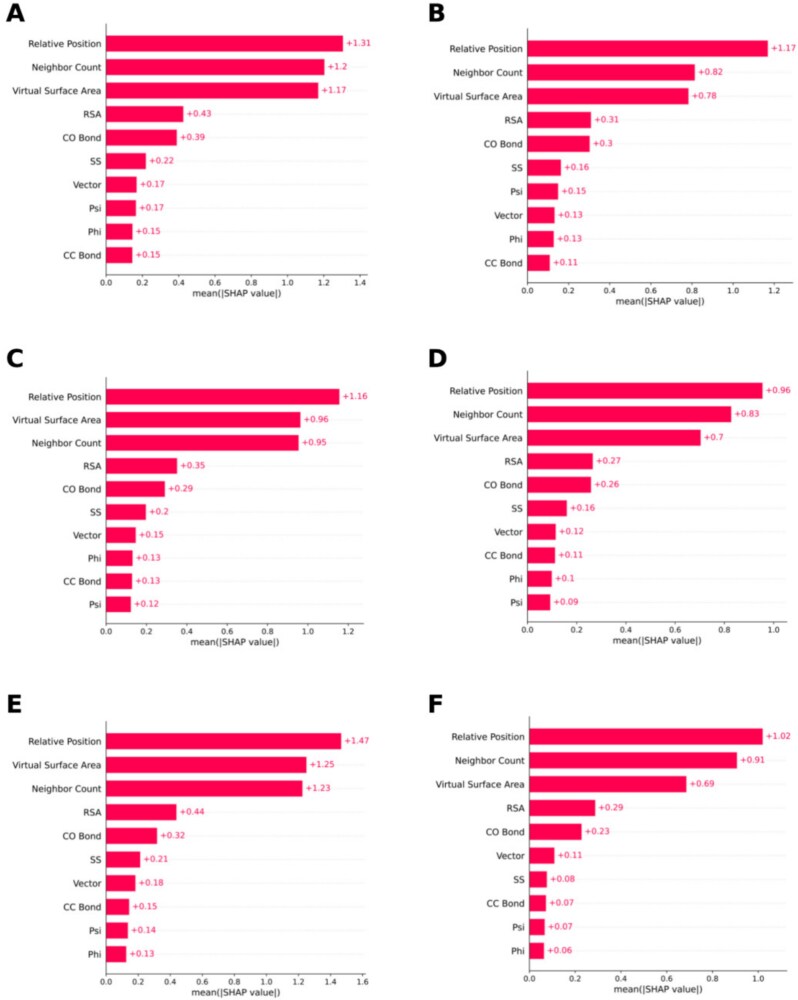
SHAP analysis results for various biological categories in the UniProt dataset, including the Animal, Archaea, Bacteria, Fungi, Plant, and Protista categories. Each SHAP bar plot was generated from the averaged absolute SHAP values of the structural features. (A) Animal. (B) Archaea. (C) Bacteria. (D) Fungi. (E) Plant. (F) Protista.

The SHAP bar plots for the RCSB and UniProt datasets are provided in Figs 8 and 9, respectively. In both figures, ‘‘Relative position’’ has always been the most important feature for all categories. For the second position, we have Virtual Surface Area and Neighbor count competing. RSA, CO bond, SS, and Vector have consistently been positioned between 4 and 7 for all categories in both figures. CC Bond, Psi, and Phi angle shuffle between the eighth and 10th positions. For most of the categories belonging to both datasets, Psi angle contributes more than Phi angle.

**Figure 10.**
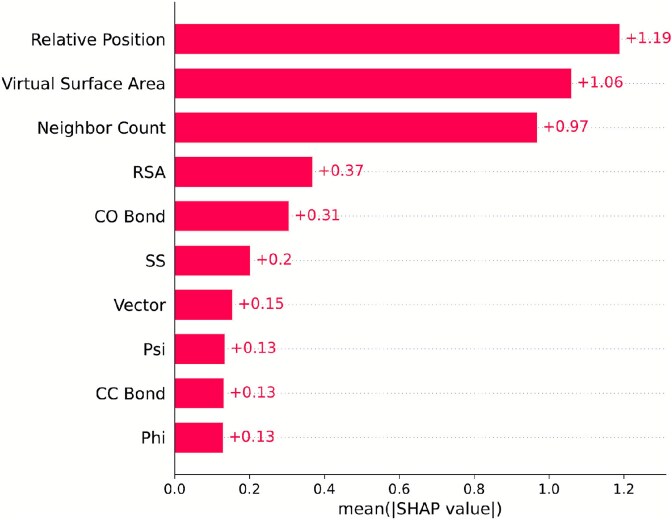
SHAP analysis for the independent dataset, providing global feature importance results. The SHAP bar plot was generated from the averaged absolute SHAP values of the structural features.

Finally, taking all samples from the independent dataset, we did a SHAP analysis on the structural features to examine global feature importance (not limited to a particular category). The SHAP bar plot is given in Fig. 10. Relative position has held the top position among the structural features, and Phi angle is the less contributing structural feature among all of them.

From the plots, we can observe that Relative Position, Virtual Surface Area, Neighbor Count, RSA, and CO Bond consistently rank among the top five across all categories in both the RCSB and UniProt datasets. Thus, across different categories, they demonstrate consistent performance.

**Figure 11.**
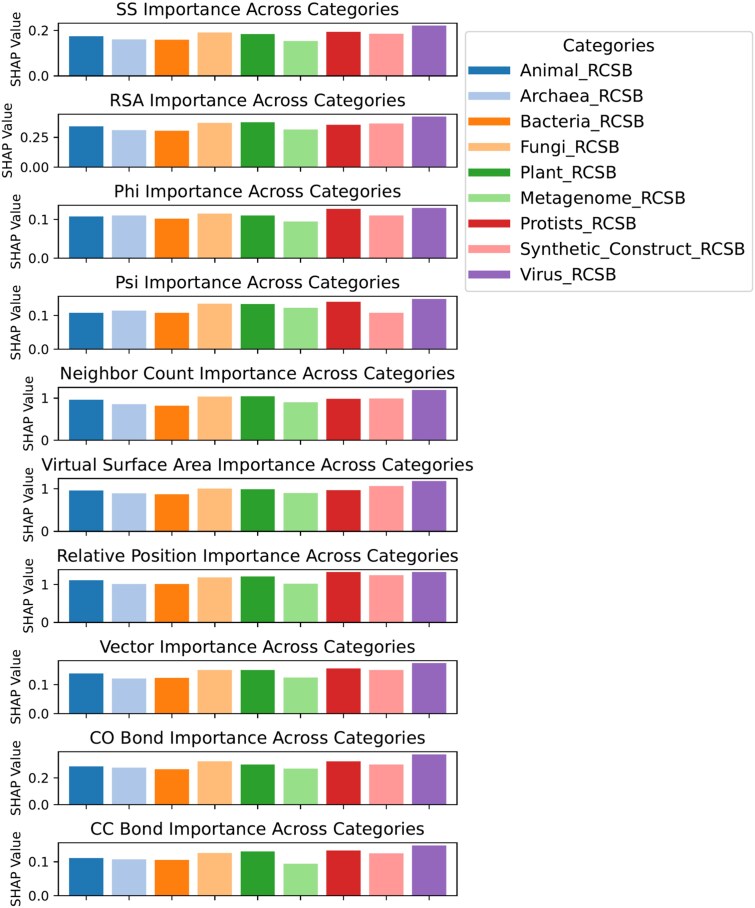
Category-wise SHAP value distribution of structural features. Bar plots were generated from the averaged absolute SHAP values of the structural features across different categories.

**Figure 12.**
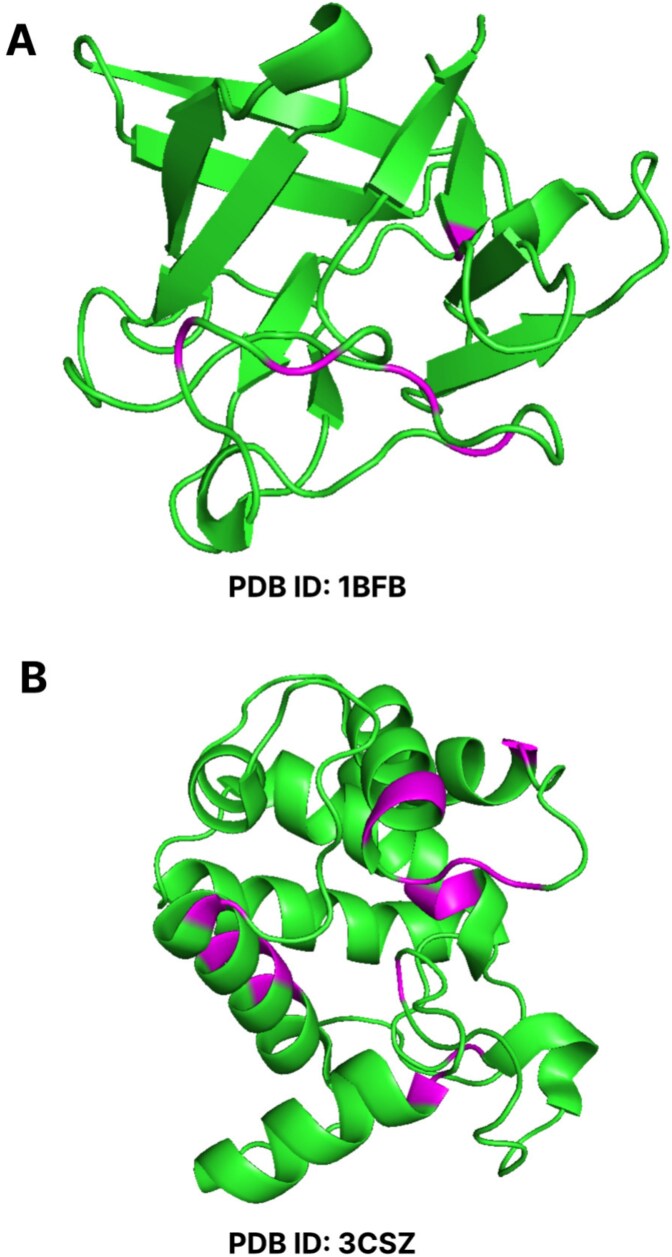
Visualizations of residue-wise SHAP values on protein structures for proteins having PDB IDs: (A) 1BFB and (B) 3CSZ, where residues with higher SHAP importance are distinguished from those with lower importance in the structural representation.

To assess how structural features contribute to the variation across categories, we analyzed SHAP values for the structural feature across nine RCSB-derived categories in Fig. 11. We did it on the RCSB dataset because it had a larger number of proteins than the Uniprot dataset. Throughout all structural features, Animals and Bacteria consistently show lower SHAP importance values than the Virus. This may indicate that in animal and bacterial proteins, the predictive contribution of individual structural descriptors is less pronounced. A probable explanation lies in the fact that animal and bacterial proteins tend to have more diverse and flexible folds, often determined by large multi-domain architectures and extensive posttranslational modifications that reduce the discriminative power of structural features [53--55]. On the contrary, viral proteins, which obtained the highest feature importance, usually bear compact, highly constrained structures optimized for capsid formation or host interaction [56, 57]. Such rigid motifs might make structural features such as SS, RSA, and Phi and Psi angles more informative and consistent for the model. Therefore, the higher structural feature importance of viruses likely reflects the reduced conformational variability of their structures compared with animal and bacterial proteins. This observation is further supported by a residue-level SHAP case study in which per-residue structural importance values were mapped onto protein structures in Fig. 12. Indeed, for the Animal protein, with PDB ID 1BFB, only five out of 124 residues ( 4%) showed high SHAP importance, but for the Virus protein with PDB ID 3CSZ, 15 of 159 residues ( 10%) were highly important according to SHAP (Fig. 12). The magenta-colored residues represent positions with higher SHAP importance in the visualization, while the green residues indicate lower importance. Therefore, this comparison confirms that viral proteins have a higher percentage of structurally important residues, which justifies that structural features in viral proteins are more influential compared with animal proteins.

### Neighborhood analysis using SHAP

**Figure 13.**
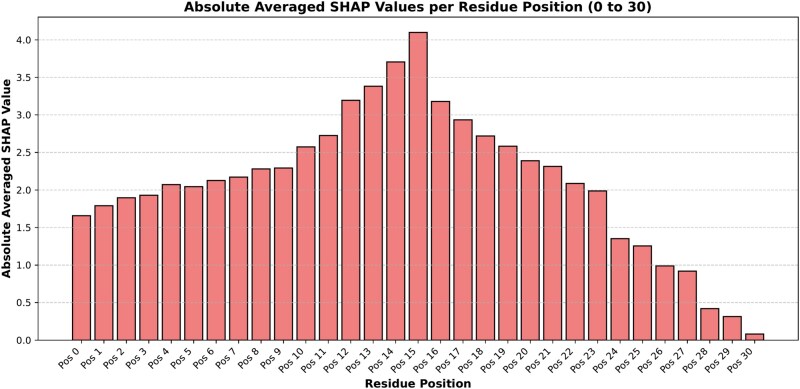
Bar plot with averaged absolute SHAP values on the y-axis and individual residue positions on the x-axis. Each bar represents the contribution of a single residue at a specific position within the 31-length window (positions 0 to 30) to the model’s prediction. The y-axis shows the absolute averaged SHAP value for each residue position, highlighting the relative importance of different positions in the local sequence context.

We utilized SHAP for feature analysis on the Word embedding, which is used as input in the ResNet branch of our final proposed model, to investigate the contribution of local residue context to the model predictions. At first, we calculated the absolute averaged SHAP values for each residue position within the 31-length window from positions 0 to 30 centered around the target site. We plotted the SHAP bar plot of this in Fig. 13. In the figure, the central target residue and especially the residues that are near the target site exhibit higher SHAP values, whereas distant residues gradually have lesser contributions to the model’s output. These indicate the influence of local context on the model’s output and suggest that residues in close sequential proximity to the prediction site carry more predictive information than those at the periphery.

**Figure 14.**
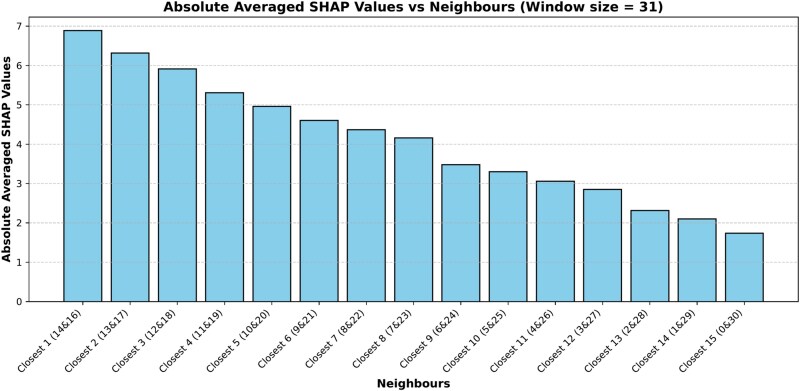
Bar plot with averaged absolute SHAP values on the y-axis and neighboring residues on the x-axis. “Closest 1 (14 & 16)” refers to the nearest residues (positions 14 and 16 within the window). Similar formats apply to other x-axis labels. The y-axis holds the summed, averaged absolute SHAP values of the symmetric residue pairs around the central residue in the window.

To further validate the significance of neighborhood residues, we conducted a pairwise analysis by summing SHAP values of symmetrical neighbors around the center. Specifically, residues sequentially equidistant from the target site (e.g. positions 14 and 16, 13 and 17,..., 0 and 30) were grouped, and their combined SHAP contributions were illustrated in Fig. 14. The bar plot reveals a declining trend in SHAP values as the pair’s distance from the target residue increases. This reinforces our previous conclusion that immediate neighbors contribute more significantly to the classification decision. The findings prove that our model learns meaningful patterns within the local residue environment, emphasizing the value of incorporating neighborhood context for improved interpretability and performance.

**Figure 15.**
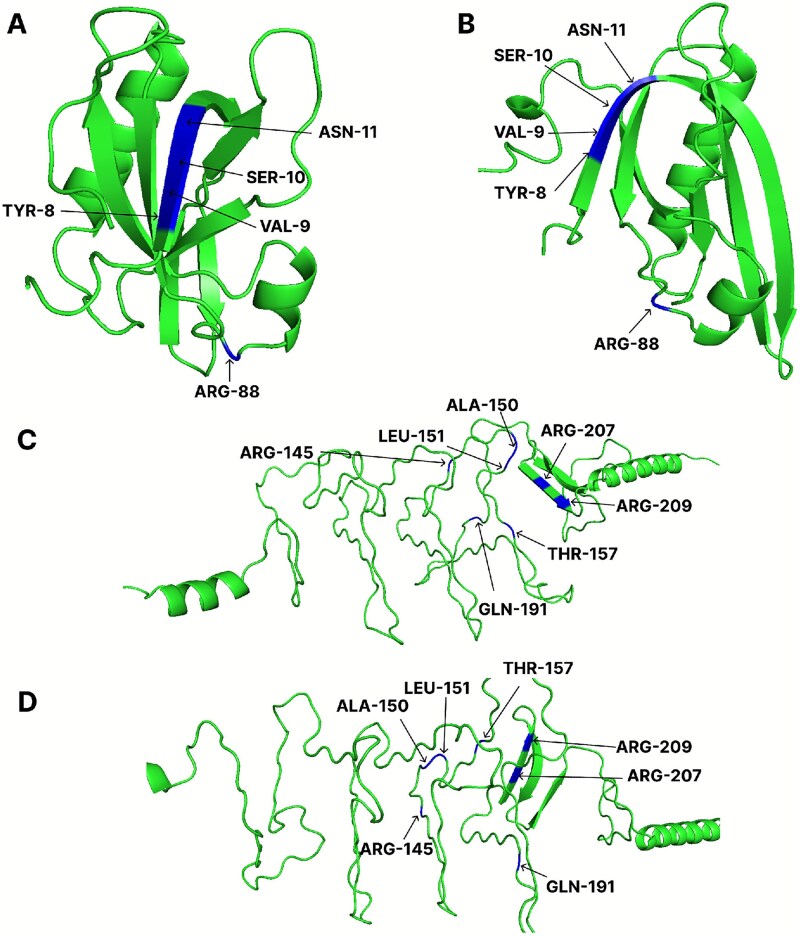
PyMOL visualization of representative positive sites that were correctly predicted only by our proposed model and were incorrectly identified by DeepGlycanSite, CAPSIF:V, CAPSIF:G, PS-G, and PS-S. The figure shows (A) PDB ID 7EE4, chain A (experimental structure), (B) PDB ID 7EE4, chain A (ESMFold-predicted structure), (C) PDB ID 6WWX, chain A (experimental structure), and (D) PDB ID 6WWX, chain A (ESMFold-predicted structure). For labeling, we used the first three letters of each residue type followed by its sequence position (e.g. ARG-88). Each subfigure displays the 3D structure of a protein: alpha helices are shown as spiral coils, beta sheets as flat arrows, and connecting regions as wavy lines or random coils.

### Case study---DeepCPBSite in diverse structural environments

We conducted a case study on four proteins with the PDB IDs 7EE4 and 6WWX in Fig. 15. In the figure, the positive sites are color-coded in blue. We collected the experimental protein structures from the RCSB database and also the structures predicted by ESMFold and visualized them using PyMOL [58]. In all four subfigures, all sites were correctly classified by DeepCPBSite. But DeepGlycanSite, CAPSIF:V, CAPSIF:G, PS-G, and PS-S could not do so. They failed to correctly predict sites 8, 9, 10, 11, and 88 of 7EE4 and 145, 150, 151, 157, 191, 207, and 209 of 6WWX. In Fig. 15, we can see that sites in positions 8, 9, 10, and 11 of 7EE4 were in the beta sheets (flat arrows) and position 88 was in the connecting region (wavy lines or random coils). The site positions 209 and 207 were in the beta sheets, and positions 145, 150, 151, 157, and 191 were in the connecting region. These observations suggest that although other SOTA methods struggled with diverse structural environments, DeepCPBSite consistently made correct predictions, regardless of the structural setting surrounding the sites.

## Discussion and conclusion

In this work, we have designed a novel ensemble model, DeepCPBSite, to predict non-covalent carbohydrate--protein binding sites. It is built with a ResNet+FNN DL architecture combining three separate models trained using three different data balancing approaches: random undersampling, weighted oversampling, and class-weighted loss. We constructed three datasets from RCSB, Uniprot, and CASP. The RCSB dataset was divided into a training set and an independent test set. From the independent test set, we created a common benchmark test set, TS53, for comparison with SOTA methods. Additionally, another test set, TS37, was derived from the UniProt dataset, and the TS14 test set from CASP.

At first, we selected the feature set for our model by three different feature selection techniques. All three feature selection techniques resulted in the same selected feature groups: ‘‘ProtT5-XL-U50, Structural, ESM-2.’’ Multiple feature selection techniques settling on the same feature set speak to the robustness of the selected feature set. The overall size of the feature set was 2340 + 31 (FNN branch + ResNet branch). A total of 1024 features came from ProtT5-XL-U50 embeddings, 36 from structural features, and 1280 from ESM-2 embeddings, summing up to a total of 2340. Lastly, 31 features came from word embedding. Although ProtT5-XL-U50 and ESM-2 are both large-scale PLMs, they differ in architecture, tokenization, and pretraining strategies. Feature selection analyses revealed that the embeddings from each PLM contributed distinct, nonredundant information relevant to our task. Empirically, combining the embeddings from both models consistently led to better performance compared with using either one alone. This suggests that the representations learned by the two PLMs capture different aspects of protein sequence semantics, and leveraging both together enhances the model’s predictive capability.

We compared the SOTA methods using both experimental structures (retrieved from RCSB) and predicted ESMFold structures. DeepCPBSite demonstrates almost consistent performance under experimental and predicted ESMFold structures, with only a 0.1%--2.4% performance drop across various metrics using predicted ESMFold structures. In contrast, the second-best model, DeepGlycanSite, showed a larger performance drop of 0%--4.2%. It proves the reliability and consistency of our model, regardless of the source of structural features. DeepCPBSite achieved 3.84% and 5.20% more MCC, 8.43% and 15.84% more AUPR, and 3.77% and 4.64% more F1 than DeepGlycanSite (the second-best model) when using experimental structures and ESMFold structures, respectively. We have outperformed all SOTA methods using a simpler downstream model than the complex GNN or transformer-based ones. DeepCPBSite, our proposed model, has only 4134048 parameters, making it nearly 20x smaller than DeepGlycanSite (80853506 parameters) and nearly 25x smaller than CAPSIF:V (102676001 parameters), while still achieving superior performance. In contrast, CAPSIF:G has just 236009 parameters, and both PS-S and PS-G have 1111154 parameters, but they lack the predictive power of our model. This demonstrates that DeepCPBSite is significantly cheaper and more efficient in downstream predictions without sacrificing accuracy. We have observed that structural features derived from ESMFold structures perform better than those from AlphaFold structures for predicting non-covalent carbohydrate--protein binding sites for Fungi and Archaea. A probable cause may be that AlphaFold is heavily dependent on preexisting genomic databases for MSA construction, but ESMFold does not require them. ESMFold leverages the power of DL and language models. Both structural features are, however, limited by the accuracy of their underlying models, and interpretation should be made with caution for proteins with sparse homologs or unusual folds. Future work may thus consider hybrid approaches or uncertainty estimation to mitigate error propagation from structural predictions. It could further investigate these differences and their impact on carbohydrate--protein interactions.

We concluded that for every category under RCSB or UniProt datasets, Relative position is the best-contributing feature among all structural features. And for most of the categories under RCSB and UniProt datasets, Phi angle contributed less than the other structural features. The probable reason may be Phi angle, while crucial for characterizing protein backbone conformation, but cannot fully provide the distinguishing factors of protein structures on its own. Structural information like neighbor count or relative distances might offer a more complete or complementary view. Future studies could delve deeper into these aspects and uncover valuable insights that enhance the understanding of carbohydrate--protein binding sites. The structural features in viral proteins are more influential compared with animal proteins. The SHAP neighborhood analysis shows how local context affects the model’s output and implies that residues near the prediction site sequentially carry more predictive information than residues farther away. The results highlight the importance of integrating neighborhood context for enhanced interpretability and performance by demonstrating that our model learns significant patterns within the local residue environment.

There are some limitations to this study. Some test proteins may have been part of the training data for the structure prediction models (AlphaFold and ESMFold), potentially introducing bias into the downstream predictions. This could lead to overestimated performance, especially for targets with strong evolutionary signals in the form of high-quality MSAs. It is worth noting that this potential source of bias has also been overlooked in several related studies. Nonetheless, for a more rigorous and fair evaluation, future work should consider benchmark datasets that are explicitly free from such overlaps or pretraining influence from structure prediction models. As future work, it would be valuable to assess the model’s generalizability to orphan proteins---those lacking sequence and structural homologs in major databases such as BFD, MGnify, UniRef, and UniClust---to evaluate its true transferability. Additionally, following the approach of DeepGlycanSite, reporting performance across different carbohydrate classes (mono-, di-, and oligosaccharides) could offer more detailed insights into the model’s strengths and limitations. Our model is likely to perform worse in situations involving protein complexes with glycosylation sites, since such complexes were excluded from the training dataset to maintain consistency with non-glycosylated protein--carbohydrate interactions. The model thus has never been exposed to the unique structural and biochemical features of binding that are mediated by glycosylation and may not be able to accurately identify such sites during prediction. This means that future expansion of the dataset needs to involve more glycosylated protein--carbohydrate complexes for better generalization.

In our study, we have built a simpler and novel model, DeepCPBSite, for predicting carbohydrate--protein binding sites. It outperformed existing SOTA methods. The python scripts for reproducing the results and datasets are provided at https://github.com/nafcoder/DeepCPBSite. The prediction of the proposed model can serve as a foundation for identifying potential carbohydrate--protein binding sites. These predicted sites can help guide the search for carbohydrate--protein binding sites for novel proteins. This could lead to a quicker discovery of new carbohydrate--protein binding sites and their functional implications in biological systems. Given its impact on several biological processes, including inflammation, signal transduction, cell adhesion, host--pathogen recognition, protein structure stabilization, and more, we hope that DeepCPBSite will make significant improvements to the field of research on the prediction of non-covalent carbohydrate--protein binding sites.

**Key Points**

- We have achieved better results than all SOTA methods for predicting protein--carbohydrate binding sites. Our proposed model is more efficient and has a simpler architecture.
- RCSB, Uniprot, and CASP are the three sources from which we constructed our datasets.
- To choose the best feature set for our prediction task, we used three distinct feature selection methods: Elastic Net, RFE, and IFS. The same feature set has been obtained by all three approaches, demonstrating the robustness of the chosen features and indicating a high degree of consensus.
- In this study, we investigated and compared five distinct imbalance handling strategies. Three of these techniques were chosen for the final ensemble model: class-weighted loss, weighted oversampling, and random undersampling.
- The structural features for various kingdoms/categories from two kinds of datasets (RCSB and UniProt) have been subjected to a SHAP analysis. We also conducted SHAP analysis on the ResNet branch to determine the impact of local residue context on model predictions.

## Supplementary Material

bbag008_Supplemental_Files

## Data Availability

The python scripts for reproducing the results and datasets used in our study are freely available at https://github.com/nafcoder/DeepCPBSite.
